# Component-Level Contributions of Retrieval-Augmented LLM Post-Processing in Streaming Anomaly Detection: A Matched-Operating-Point Case Audit

**DOI:** 10.3390/s26165246

**Published:** 2026-08-19

**Authors:** Changwon Baek

**Affiliations:** Department of Data Science, Cheongju University, Cheongju 28503, Republic of Korea; baekcw@cju.ac.kr

**Keywords:** anomaly detection, retrieval-augmented generation, large language models, industrial IoT, sensor streams, evaluation, measurement critique, reproducibility, point adjustment

## Abstract

Retrieval-augmented large language model (LLM) post-processing reportedly improves anomaly triage over streaming industrial Internet of Things (IoT) sensor data, yet its LLM and retrieval contributions are rarely separated. We audit a build-verified pipeline (detector, LLM, retrieval, reranking), adding each stage at a matched detector recall of 0.9 under SHA-256-frozen preregistration, with bootstrap intervals and two generator sizes on 40 NAB and SKAB streams (19 industrial). Retrieval, the preregistered primary contrast, is null in seven of eight design configurations, but a preregistered grid of lower recall targets breaks that null on precision at both targets under stream-level resampling and in two of four cells when benchmark families are the unit. Across that grid, the LLM gain (72B F1 +0.0502) shrinks as candidate recall falls (+0.0227 at 0.7, undetectable at 0.5), which is a bound, not a dose–response curve. There, retrieval buys precision at a significant cost of recall. On the 19 industrial streams, the LLM gain is null at one label-free point and exactly zero at the other two, where the generator confirmed every candidate. Faithfulness, by local natural language inference, is low in every arm and not raised by retrieval. Three annotators labeling 133 claims (Fleiss’ κ = 0.555) placed the shortfall in the verdicts; none entailed by consensus. The protocol is released as a tested artifact.

## 1. Introduction

Industrial Internet of Things (IoT) sensor streams are a primary target for online anomaly detection, and a fast-growing body of work is layering large language models (LLMs) on top of conventional detectors. In these designs, an LLM re-examines each flagged event, optionally conditioned on retrieved historical cases, to confirm or suppress the alarm and to produce a natural language explanation. This retrieval-augmented generation (RAG) formulation, which conditions a parametric generator on a non-parametric external memory, was introduced for knowledge-intensive language tasks [1]. Section 2.1 reviews the gains later reported for log analysis, time-series detection and industrial diagnostics. What this line of work shares, almost without exception, is a positive-result framing built on single-benchmark, single-pipeline comparisons that report an aggregate gain while leaving the LLM component’s contribution and the retrieval component’s contribution fused into one number.

This aggregation hides the question a practitioner actually faces: how much of the reported improvement comes from the LLM re-examining the event and how much from retrieving past cases? The two are rarely separated, and almost never at a matched operating point. The problem is compounded by how streaming anomaly detection is scored. The widely used point adjustment protocol can make even a random score appear strong [2], so an aggregate comparison run at unequal or arbitrary thresholds can inflate a component’s apparent value. The priors that motivate adding retrieval and reranking in the first place (more retrieved context and better-ranked context improve generation) are established largely in resource-rich open-domain question answering (QA), and their transfer to low-resource industrial anomaly triage has not been tested component by component.

We therefore audit, rather than optimize, a *constructed reference pipeline*. We do not re-audit any specific published system; their tasks, data, and architectures differ, so their reported numbers are not on trial here. Instead, we build a reference pipeline that instantiates documented RAG practices as a four-arm ladder (detector, then +LLM, then +retrieval, then +rerank) and ask what each stage actually adds. The ablation runs in the additive rather than the usual subtractive direction, so that every configuration is a system someone might actually deploy. Each stage’s *increment*, the difference between two adjacent configurations, is evaluated at a single matched operating point (a fixed detector recall of 0.9), so threshold choice cannot differ between configurations. Uncertainty on every increment is quantified by stream-level bootstrap confidence intervals in a design frozen by SHA-256 hash before any results are computed, including a preregistered rule for reporting a null on the primary retrieval contrast. The audit runs with two generator sizes (7B and 72B parameters), and explanation faithfulness is scored with a local natural language inference model, deliberately excluding any externally hosted LLM as judge. Preregistered build integrity checks first confirm that the generator and retrieval stages are genuinely functional rather than templated stand-ins.

The findings are a measurement critique. The LLM increment is real but modest and precision-weighted at both generator sizes. The retrieval increment, the preregistered primary contrast, is an honest null for detection quality; its F1 and precision confidence intervals include zero at both generator sizes. At 72B, this is a bounded null; the interval excludes benefits larger than +0.023 F1, which is less than half of the +0.050 that the LLM stage delivers. At 7B, the study is too weakly powered to bound anything, and we distinguish the two throughout (Section 4). Retrieval is not inert; it significantly degrades recall on the NAB series. Adding cross-encoder reranking significantly worsens F1 and precision with the 72B generator in the frozen configuration, but that effect does not reproduce under any of the nine configuration changes in which the reranking arm is later run, so we withdraw it rather than draw a boundary condition from it. Explanation faithfulness does not rise with retrieval, and three independent annotators judging 133 claims (Fleiss’ κ = 0.555) locate the shortfall: claims restating the measurements are often entailed, but none of the 64 that deliver the system’s verdict is.

Sensitivity axes examined at revision sharpen this. The retrieval null holds under every change of detector, retriever, corpus/eval split and generator family, and for F1 under every neighbor count, but not across the operating point. At label-free thresholds, retrieval reverses to a loss; at the two preregistered fixed-recall targets, it turns significantly positive for precision, breaking the preregistered null there, while F1 remains null and recall remains negative. What the audit finds is therefore not that these components are inert but that their measured value depends on where the operating point is set. Where the candidate set is tightened under the preregistered grid, retrieval buys precision at the cost of recall; at the label-free points a deployment can actually reach, it forfeits recall without buying precision, and the generator’s F1 gain falls towards zero.

That finding is the paper’s first contribution. The second is the instrument that makes such a finding checkable: a reusable, preregistered decomposition protocol whose build integrity gate verifies that the retrieval and generation stages are genuinely functional before they are measured, released as a tested package. The conclusions come from a single-configuration case study (40 streams; NAB and SKAB), and we bound their scope accordingly (Section 5.4).

## 2. Related Work

### 2.1. LLM- and RAG-Based Anomaly Detection: A Pattern of Optimistic Framing

Retrieval-augmented generation was originally proposed to supplement the limits of parametric knowledge with a non-parametric external memory [1] and has since spread into anomaly detection across logs, industrial sensors and time series, mostly reporting sizeable aggregate gains. In log analysis, vector database and token–template frameworks [3,4] are joined by EagerLog, which reported an F1 of 93.65% from roughly ten active learning-labeled log sequences, 15.32% higher than existing methods [5]. In industrial predictive maintenance, RAAD-LLM raised accuracy from 70.7% to 88.6% on plastic manufacturing shop floor data relative to the authors’ own earlier model and was also validated on the SKAB [6]. Outside industrial settings, pairing autoencoders with retrieval improved recall and interpretability on credit card fraud data [7]. In time-series anomaly detection, RATFM attained performance comparable to in-domain fine-tuning without domain-dependent training, by retrieving examples from the same domain at inference [8], and TSAD-RAG reported F1 gains of 17% and 5% on a standard synthetic benchmark and on a dataset of its authors’ own construction [9]. Further work covered clustering-informed retrieval sampling, LLM-assisted control chart labeling, repair manual-augmented factory systems and frozen core retrieval [10,11,12,13], alongside retrieval-free zero-shot and distillation uses of LLMs [14,15].

That is the gap this study targets. We do not re-audit the systems above, whose tasks and data differ; we build a representative pipeline instantiating these documented practices and decompose it through a four-arm ladder (detector, +LLM, +retrieval, +rerank) matched at a common operating point.

### 2.2. Evaluation Methodology Critiques in Time-Series Anomaly Detection

The direct methodological ancestor of this audit is the demonstration, both theoretical and empirical, that the point adjustment (PA) protocol can make even a random anomaly score appear to be state-of-the-art [2]. The concern recurs throughout the threshold dependence literature. According to one review, PA can inflate performance so severely that a random guess outperforms all algorithms. Separately, under more robust protocols, that review found that a simple principal component analysis (PCA) baseline outperforms many recent deep learning methods [16]. A cross-comparison of 17 algorithms examined how threshold and metric choices affect measured performance [17]. Remedies range from a self-supervised tri-domain alternative [18] to a decay function correction [19]. Threshold dependence has also been challenged directly: the Volume-Under-the-Surface family describes itself as “parameter-free, threshold-independent” because it integrates accuracy across all thresholds and buffer sizes rather than fixing one [20,21]. A semantics-aware metric partitions each anomaly’s local region into three detection semantic subregions to address point-level bias and near-miss insensitivity and integrates detection quality across the whole threshold spectrum to remove threshold choice inconsistency [22], while an Operator Interest metric works at a fixed threshold and instead reconciles point-wise with event-wise evaluation [23]. Industrial multivariate detection shows the same pattern. A reproduction study confirmed that favorable point-level performance need not imply strong event-level detection [24], and event-level re-evaluation with counterfactual explanation reconfirmed that point-adjusted scores can look far higher than strict event-level measurements [25]. Score mapping itself can cause PA metrics to overestimate performance under certain mapping strategies [26]. An independent adversarial stress test extended the critique further, concluding that existing corrections are only partial once best-of-N reporting is allowed. Under repeated runs, affiliation-F1 and receiver operating characteristic (ROC) family metrics remain gameable, precision–recall (PR) family metrics and PA%K are comparatively robust and, on the NAB, even VUS-PR can be gamed at a large N [27].

Our fixed-recall matched operating point protocol starts from the same concern as this line of work critiquing thresholds, that a single arbitrary threshold can determine conclusions, but responds to it differently: it isolates LLM versus retrieval increments rather than contesting the gameability of any one metric. The finding that VUS-PR remains gameable on the NAB under best-of-N reporting [27] supports this choice.

### 2.3. RAG Faithfulness and Groundedness Evaluation

Faithfulness evaluation has moved from language model (LM) judge scoring—ARES scores context relevance, faithfulness and answer relevance with automated judges [28], surveyed since alongside retrieval- and generation-specific metrics [29,30] and eRAG, which shows that document-level relevance labels correlate only weakly with downstream performance and proposes downstream-grounded labeling instead [31]—toward a reflective distinction between correctness and faithfulness. Citation correctness alone is insufficient because a model may cite a document without having used it as grounds for its answer, a post-rationalization that one study finds in up to 57% of citations [32]. Further work models unfaithfulness as a retrieved–parametric conflict at the fact level [33]. Another builds multilingual faithfulness benchmarks [34], and an agentic search study reports that optimizing for final answer accuracy alone leaves substantial room to improve intermediate reasoning faithfulness [35]. Closest to the present domain is a recent audit of epistemic overreach in LLM-generated explanations of personal sensing data, explanations that can “sound coherent and personally meaningful even when the underlying evidence is sparse, incomplete, or missing” [36].

Most of this line of work scores faithfulness with an external LLM-as-judge. We instead compute it by local NLI entailment, excluding external LLM generation or judgment on principle. What we find (SQ4, the faithfulness question defined with the other four in Section 4) aligns with both audits above [32,36] and raises the same concern regarding streaming industrial triage.

### 2.4. Retrieval Robustness and Small-Model Vulnerability

The “lost-in-the-middle” phenomenon—performance degrading sharply when the needed information sits in the middle of a long context even though it is present—was first demonstrated in multi-document QA and key value retrieval [37]: availability does not guarantee use. This irrelevant context can harm retrieval-augmented LM performance and was characterized across five open-domain QA benchmarks, with multi-hop reasoning being the most exposed. NLI-based filtering of non-entailing passages prevents the degradation but discards relevant passages too [38]. Most directly relevant is a controlled study across five model scales (360M–8B) and three architecture families in which models of 7B parameters or smaller failed, even under oracle retrieval, to extract the correct answer in 85–100% of cases on questions they could not already answer parametrically, and added context corrupted 42–100% of previously correct answers [39].

This literature is drawn largely from QA and reasoning tasks in which small models fail to exploit, or are actively harmed by, retrieved context. Whether the same mechanism operates in industrial streaming triage is an empirical question, and our exploratory SQ5 initially appears to answer it in the affirmative; however, the sensitivity analyses do not sustain that reading, and Section 5.2 sets out why we withdraw it.

### 2.5. Downstream Effects of Reranking

That reranking improves downstream RAG quality is a well-documented prior. A controlled evaluation of nine retriever–reranker combinations concluded that “LLM-based reranking consistently improves downstream generation quality”—a comparison between reranker families rather than against a no-reranking baseline [40]. The surrounding literature builds on that assumption, deploying reranking as a component of utility-optimized [41], recursive [42] and hybrid [43] pipelines and standardizing such comparisons in a shared evaluation platform [44]. A controlled comparison of vector, hybrid and reranked retrieval qualifies that reranking helps one generator and slightly hurts another [45].

This assumption already shows cracks within the same line of work. Comparing four retrieval pipelines in medical question answering, rerank-only was optimal for the accuracy–latency trade-off, but cascading reranking with another retrieval stage introduced noise and yielded no additional benefit [46]. Reranking is not unconditionally beneficial; its gain attenuates or reverses depending on pipeline position and combination.

This prior is established mainly in resource-rich text corpora and open-domain QA. Our SQ3 observes the opposite sign under narrow conditions (industrial streaming triage, a 72B generator, one bge reranker configuration). Our arm is rerank-only in that taxonomy, that study’s preferred default, so the observation runs against its result rather than echoing its cascading caution [46]. The effect does not survive our sensitivity checks, and we withdraw it rather than treating it as a boundary condition on the prior.

### 2.6. Positioning of the Present Study

These five strands have accumulated largely independently on different benchmarks and under different methodological conventions. Each is individually robust, yet we found no study that applies all five controls together to a single end-to-end pipeline at a matched operating point separating the LLM increment from the retrieval increment in an industrial streaming anomaly detection setting with the pipeline’s functionality verified before measurement. Because this is an absence claim, its basis is recorded rather than asserted. Every anomaly detection system cited in Section 2.1 (thirteen, excluding the RAG formulation itself) is coded in Table S19 against the two controls this study turns on. Of the thirteen systems coded in Table S19, we hold eight in full text. Five report a component-wise contrast, and in none of the five could we locate a common operating point across the contrasted arms. The two that name their arms explicitly sit at a different recall (0.68 against 0.89; 0.80 against 0.92). The remaining five were available only in abstract and are left undetermined since an abstract cannot establish that a study did not do something. This gap defines the scope of what follows; no claim here rests on being first to fill it. We combine the five strands in a single preregistered four-arm decomposition with bootstrap confidence intervals and offer the matched operating point protocol with its build integrity suite as a reusable release artifact rather than a claim of best practice or state-of-the-art performance.

## 3. Methodology

### 3.1. Data

Evaluation combines two public streaming anomaly benchmarks: the real-world (non-artificial) data categories of the Numenta Anomaly Benchmark (NAB) [47,48] and the official Skoltech Anomaly Benchmark (SKAB) master release [49]. Both are used in their official, unmodified form. No synthetic anomalies are injected. The detector baseline (Section 4) is computed on the full pooled set of 80 series, reporting the area under the precision–recall curve (AUPRC) together with a fixed-recall grid of F1 and point-adjusted F1 (PA-F1). No retrieval is involved at this stage, so the full pool is usable. The arm comparison (Section 4), by contrast, uses only the 40-series eval half of a disjoint 80-series corpus/eval partition described below. Corpus set series serve only as the retrieval source and are structurally excluded from evaluation. This restriction also keeps the required per-event LLM generation footprint tractable: 896 labeled anomalous events on the eval set yield 5376 LLM decisions (896 events × 3 LLM-based arms × 2 generator sizes). All series-to-corpus and series-to-eval assignments are fixed under seed 42 and frozen prior to any result computation (see Preregistration below).

Both benchmarks have known structural limits: the NAB is largely univariate and the SKAB’s sequences are short. The choice therefore needs defending. It follows from what the design requires. The audit needs *many labeled series*, not long ones. The retrieval corpus is built from past anomaly cases, so a disjoint corpus/eval partition demands labeled segments spread across many independent series. The NAB and SKAB together supply 80, split 40/40. Large multivariate process benchmarks such as SWaT or WADI offer richer sequences but far fewer independent series, leaving the retrieval corpus either tiny or overlapping with evaluation. The measurand is also an increment at a shared candidate set rather than absolute detection quality, so benchmark difficulty shifts the absolute numbers (AUPRC differs substantially between the two; Section 4) without undermining a comparison between arms consuming identical candidate events. The corpus half yields 78 retrievable cases, one per labeled anomaly segment, so, with the same-series guard, a k = 5 query returns about 6% of the bank. The null below is conditional on a case bank of that size. Using both in their official, unmodified form with no injected anomalies is itself part of the preregistered reproducibility claim.

The two halves are not the same kind of data, which matters for how far the result travels. Of the 40 evaluation streams, 19 are SKAB industrial process series. The remaining 21 are NAB series covering, in the categories as distributed in the NAB corpus [48], cloud metrics (10), road traffic (4), tweet volume (4), ad exchange (2) and 1 ambient-temperature trace with a known system failure. Only the SKAB half and the traffic and machine failure traces are physical sensor streams in the sense this journal’s readers would mean, so we report the primary contrast stratified by family as well as pooled (Section 4.3) rather than allowing a pooled number stand for industrial sensing.

The cost of this choice is external validity, and we state it rather than argue it away. Our conclusions are established for these two benchmarks and do not transfer by assumption to modern large-scale multivariate suites. Applying the same decomposition to such benchmarks is the most direct extension of this work, and the released protocol is written to make that a configuration change rather than a reimplementation.

### 3.2. Constructed Reference Pipeline

The system is a four-stage pipeline that instantiates practices documented in prior retrieval-augmented generation (RAG) work rather than a bespoke architecture. We refer to it as a *constructed reference pipeline* to avoid implying it is representative of all deployed systems.

Two things should be said about how the components below were selected, because an audit whose components were chosen to produce a result would be worthless: First, none of them was tuned. Every algorithm, hyperparameter and model identifier was fixed and SHA-256-frozen before any result was computed, and the preregistration explicitly prohibits adjusting any of them in response to observed audit outcomes. Each choice is a documented default rather than a search outcome: IsolationForest as a standard deterministic unsupervised detector; bge-large and bge-reranker-base as widely used open embedding and cross-encoder models; k = 5 as the common default neighbor count; Qwen2.5-Instruct as an open-weight instruction-tuned family available at two sizes under identical serving conditions. Citations are given only where a choice follows published evidence rather than convention.

Second, and more usefully than any argument of intent, we subsequently vary these choices and measure what happens. The sensitivity analyses in Section 4.7 replace the detector, the retriever, the neighbor count, the corpus/eval split and the generator family in turn, and the preregistered primary conclusion is unchanged in seven of the eight resulting configurations. Separately, three label-free operating points change it, and a preregistered fixed-recall grid isolates why. We report both as findings rather than caveats.

1. **Detector.** An IsolationForest (n_estimators = 200, random_state = 42) operating on the raw value plus causal (backward-only) rolling mean and standard deviation (window = 100) produces a continuous anomaly score that is fully deterministic given the seed. Candidate events for downstream triage are generated at a single recall operating point of 0.9 (see Operating Point framework, below).
2. **Retrieval corpus.** The pooled 80-series set is randomly partitioned (seed 42) into two disjoint 40-series sets, a corpus set (the source of retrievable historical anomaly cases) and an eval set, so that retrieved neighbors for any eval series query are drawn only from entirely different corpus set series, never from the queried series itself. A per-query exclude series guard provides a redundant safeguard. Candidate windows are embedded with BAAI/bge-large-en-v1.5 and indexed with exact (brute-force) cosine similarity, a deterministic nearest-neighbor procedure. The default retrieval arm returns k = 5 neighbors. The reranking arm retrieves a pool of 10 candidates and reorders them with the cross-encoder BAAI/bge-reranker-base before truncating to the top 5. Inclusion of a reranking stage follows the documented evidence that reranker choice materially affects downstream RAG quality [40].
3. **Generator.** Two open-weight instruction models, Qwen2.5-7B-Instruct and Qwen2.5-72B-Instruct, are served as Q8_0 GGUF quantizations via llama.cpp with CUDA (sm_121) using greedy decoding (temperature = 0, seed = 42) for full determinism. Conditioned on the current window’s detector output and, where applicable, the retrieved historical context, the generator issues a confirm/suppress decision on each candidate event plus a natural language explanation. This confirm/suppress-with-explanation design follows the general retrieval-augmented generation formulation wherein a parametric generator is conditioned on non-parametric retrieved memory [1].
4. **Faithfulness scorer.** Each generated explanation is decomposed into claims and scored against the concatenation of the window description and any retrieved context using a local natural language inference (NLI) cross-encoder (nli-deberta-v3-base). The reported faithfulness value is the mean entailment probability across claims. No external LLM is used for judgment at any stage, consistent with the project’s exclusion of externally hosted generation or evaluation models.

### 3.3. Experimental Arms and Increments

The ablation compares four pipeline configurations, which we call *arms*: detector_only (raw detector output, no LLM), llm (detector candidates confirmed or suppressed by the generator, no retrieval), llm_retrieval (adds the k = 5 retrieval stage) and llm_rerank (adds cross-encoder reranking of the retrieval pool). Each arm is a complete pipeline. Three *increments* are defined as pair-wise differences between adjacent arms: the **LLM increment** = llm − detector_only; the **retrieval increment** = llm_retrieval − llm; and the **reranking increment** = llm_rerank − llm_retrieval. All increments are computed de novo on the 40-series eval set and are never inherited from prior analyses. Uncertainty is quantified by stream-level bootstrap resampling (B = 10,000, seed 42), producing 95% confidence intervals (CIs) for precision, recall, F1 and PA-F1 per increment and per generator size. Each interval is computed in exactly one place and read from that source wherever it recurs; therefore, a quantity carries identical bounds in the main text, the supplementary tables and the released artifact. In the stratification analysis, the bootstrap seed is additionally derived from the identity of the quantity being resampled, so its intervals do not depend on the order in which subsets are computed.

### 3.4. Operating Point Framework

Arm comparisons are conducted at a single, matched candidate generation operating point. The detector threshold is calibrated per stream from ground-truth labels to a target recall of 0.9 (oracle thresholding, standard practice for fixed-recall evaluation), and every arm downstream of the detector inherits that stream’s candidate set. This eliminates threshold choice as a confound between arms. The preregistration fixes a grid of matched recall targets {0.5, 0.7, 0.9} for the triage arms. At submission only, the 0.9 point is executed, and increments are reported there alone. The 0.7 and 0.5 points are computed for the detector baseline only, to characterize threshold-independent behavior. At revision, we execute the arms at those two remaining points (Section 3.7). Increments are never averaged across the grid, and the confirmatory verdict remains defined at 0.9, which the freeze designates separately from the grid as the study’s candidate_generation_recall. The choice of that point as confirmatory is therefore preregistered rather than made after the results are seen.

### 3.5. Evaluation Metrics

For the detector baseline, the primary threshold-independent metric is AUPRC, reported alongside F1 and PA-F1 at each point of the fixed-recall grid. For arm comparisons, precision, recall, F1 and PA-F1 are reported at the matched 0.9 operating point. Both raw (point-wise) and point-adjusted variants are reported because the PA protocol, while standard in the time-series anomaly detection literature, can substantially inflate apparent detection performance when applied uncritically [2]. Reporting both allows the reader to gauge how much of any effect is protocol-dependent. The preregistered primary metric set (AUPRC for the detector; precision/recall/F1/PA-F1 for arms) does not include the Volume-Under-the-Surface (VUS) metric family proposed for range-based time-series anomalies [20]. VUS-PR is excluded by the preregistration and is not computed in this study.

Two inferential safeguards accompany the increment CIs. Because the primary retrieval increment result is a null, we report the minimum detectable effect (MDE) at 80% power (two-sided α = 0.05) from the bootstrap standard error (SE), so the null is expressed as a bound on undetectable effects rather than a claim of exact zero. Additionally, we apply Benjamini–Hochberg control of the false discovery rate (α = 0.05) to two-sided percentile-bootstrap *p*-values, obtained by inverting the same stream-level resampling distribution about zero and floored at 1/B, across the 26 secondary increment tests, excluding the preregistered primary contrast (the retrieval increment on F1 and precision at both generator sizes). The preregistration names precision *and* F1 as primary without specifying how to handle multiplicity within those four tests, so we report all four and do not adjust among them. Adjusted *p*-values are given for effects that depend on the correction. Neither safeguard is in the frozen preregistration, nor is the *bounded*/*undetected* grading of nulls in Section 4.7; all three are specified at revision, after the results have been seen. Because the candidate threshold is calibrated per stream from ground-truth labels (Section 3.4), realized detector_only recall is 0.978 rather than exactly 0.9. Event merging with gap = 5 and minimum length 3 lifts it above the target. Absolute precision/F1 levels are therefore optimistic relative to a deployed system without label access (Section 5.4).

### 3.6. Preregistration, Build Integrity and Reproducibility

The full experimental design (script hashes, model identifiers, quantization, decoding parameters, retrieval configuration, arm and increment definitions and the decision rule for interpreting the primary retrieval increment result) is frozen via SHA-256 hashing (commit c8f17ec) before any full-run results are computed. The freeze is anchored by the author’s own repository history and by the Zenodo deposit of that history, not by an independent registry, so its ordering relative to the runs is self-certified in the same sense as the amendment’s. The freeze includes a preregistered decision rule (Alt-2): if the 95% bootstrap confidence interval for the retrieval increment on the primary contrast includes zero at the primary operating point, the result is reported as an honest null rather than reframed post hoc. The freeze names that contrast “precision/F1” without stating whether both intervals must include zero or whether either suffices, and the two readings differ at the lower recall targets. We adopt the stricter one—the null is preserved only when both do—and apply it uniformly (Section 4.7). It is the reading under which our own preregistered headline is the easier to lose. At the frozen operating point of recall 0.9, both intervals include zero, so the headline holds either way. At the two lower preregistered targets, the precision interval excludes zero, which breaks the null under the stricter reading and leaves it standing under the looser one. We report that result under either reading, because withholding it would be the narrowing this paper criticizes. A further preregistered constraint prohibits tuning any system hyperparameter in response to observed audit outcomes. Three build integrity checks (C1–C3) are specified to run before any result is computed; Section 4.1 reports them. All scripts, the build verification test suite and the frozen configuration are released as part of the study’s artifacts (https://doi.org/10.5281/zenodo.21487221).

### 3.7. Sensitivity Analyses Added at Revision

The design above was frozen before any results were computed and remains the confirmatory record. Review noted that single choices of detector, embedding model, neighbor count, generator family and corpus/eval split leave open whether the conclusions are stable to them. We therefore examined seven sensitivity axes beyond the frozen one, yielding thirteen configurations: eight that change the pipeline’s design and five that change only the operating point at which the arms are compared. Six axes were added after the fact and written into an amendment file before each was run; the seventh, the fixed-recall grid (C-8), was preregistered in the original freeze. The strength of that record differed across them, and we separated it rather than averaging it. Five axes and the first alarm rate were declared 8 min 34 s before the campaign launched, with a tamper-evident commit preceding every result they produced. The two further alarm rates were appended on 5 August 2026 after the first label-free result was known and were fixed to bracket it symmetrically before either was run. They carry a timestamp in the amendment history but no separate commit anchor. The fixed-recall grid is different again: those operating points were preregistered in the original freeze and merely left unexecuted. What is post hoc there is the decision to run them, taken after the frozen and label-free results were known, not the choice of what to run. We use *revise* in one sense only: an axis revises the preregistered result if it changes the verdict at the frozen operating point, where the preregistered decision rule applies. No axis does that, and none was dropped after its outcome was seen. The exploratory axes narrow the conditions under which that verdict holds; the fixed-recall grid does more, breaking the preregistered null on precision at both of its targets (Section 4.7), and we report it as such rather than filing it as an exception.

Each axis varies one factor against the frozen baseline: C-1 sets the candidate threshold without labels, at a fixed alarm rate of 0.05 chosen a priori as an operational triage budget, in place of the oracle fixed-recall threshold. C-7 extends that axis to alarm rates of 0.02 and 0.10, so the label-free result does not rest on a single arbitrary budget. Their preregistration status is set out in Section 3.7. The remaining axes each replace one component against the frozen baseline: neighbor count k = 1 and 10 alongside the frozen k = 5 (C-2), the retriever with gte-large (C-3), the detector with the causal rolling z-score (C-4), a second generator family of Llama-3.1-8B and Llama-3.3-70B alongside Qwen2.5 (C-5) and two further corpus/eval splits at seeds 43 and 44 (C-6).

C-1, C-4 and C-6 change the candidate set itself, so every arm is regenerated under them. C-2 and C-3 change only what is retrieved, leaving the candidate set and the no-retrieval arm untouched, so that arm is reused from the frozen run. The reuse is verified rather than assumed; re-running the no-retrieval arm on a subset reproduced all 62 comparable decisions bit-identically after an injection control confirmed that the check was able to fail.

The six exploratory axes are reported descriptively, and bootstrap intervals are given throughout, with no multiplicity correction applied across axes and no confirmatory claim resting on them. The fixed-recall grid is not in that group (Section 4.7).

### 3.8. Computational Environment

Experiments ran on two NVIDIA DGX Spark nodes, each built on the GB10 Grace Blackwell superchip (NVIDIA Corporation, Santa Clara, CA, USA) (compute capability 12.1, 119 GB unified CPU–GPU memory, driver 580.159.03). Each node hosted the full stack locally; both generator sizes were served by llama.cpp (build 178a6c4) as OpenAI-compatible endpoints, both at Q8_0 quantization with a 4096-token context window and all layers offloaded to the GPU, together with the embedding model, the cross-encoder reranker and the NLI scorer. Parallelism was over data, not over pipeline stages. The evaluation streams were partitioned between the two nodes, each of which independently ran every arm and both generator sizes on its own shard, with no distributed reduction. All stochastic components (detector fitting, decoding, bootstrap resampling) were fixed under seed 42 to ensure deterministic, reproducible results.

## 4. Results

Results are organized by the five study questions fixed in the preregistration. SQ2, the retrieval increment, is the primary contrast.

### 4.1. Build Integrity

Before any full-run results were computed, three preregistered build integrity checks confirmed the constructed pipeline was free of the failure modes that invalidated the legacy analysis:

- The generator produced genuine, non-templated completions (C1).
- Retrieval returned distinct, non-trivial neighbor sets across queries with high but non-identical similarity (mean cosine 0.951, C2).
- Retrieved context caused measurable, non-trivial changes in generator output relative to a no-retrieval condition in all 15 probed cases (C3).

All checks passed (build verification suite; see Data Availability Statement).

### 4.2. SQ1: LLM Post-Processing Increment

At the matched recall = 0.9 operating point, the LLM increment (llm − detector_only) is positive and statistically distinguishable from zero for both generator sizes. For the 7B generator, F1 improves by 0.011 [0.005, 0.019] and precision by 0.008 [0.004, 0.013]; for the 72B generator, the improvement is larger, with F1 +0.050 [0.028, 0.078] and precision +0.042 [0.025, 0.064]. Both generator sizes pay a small but significant recall cost (7B: −0.008 [−0.015, −0.003]; 72B: −0.033 [−0.073, −0.008]), consistent with the generator suppressing some true detector-flagged events along with false positives. Table 1 reports arm-level performance and Table 2 the increments with their intervals.

Two qualifications belong with this result, both established in Section 4.7. Absolute arm-level precision and F1 here reflect the oracle, label-calibrated threshold and are optimistic relative to a label-free deployment. The increments are affected less, being differences at a shared candidate set, but are affected too. More consequentially, the LLM increment is itself conditional on the operating point; under a fixed 5% alarm rate, it is null at both sizes (7B −0.002 [−0.005, 0.000]; 72B −0.005 [−0.012, 0.001]). Across the three label-free rates, the operating point governs the size of that gain, not its existence (Section 4.7). It is robust to generator family; a second family reproduces it at both sizes.

Per-stream reporting sharpens the picture. The 72B mean of +0.050 sits well above the median of +0.025. Of the 40 streams, 30 improve and 3 worsen, and removing the 5 largest contributors still leaves +0.025. The effect is therefore broad-based rather than driven by a handful of streams, but the mean overstates the typical stream.

### 4.3. SQ2 (Primary): Retrieval Increment

**Figure 1:**
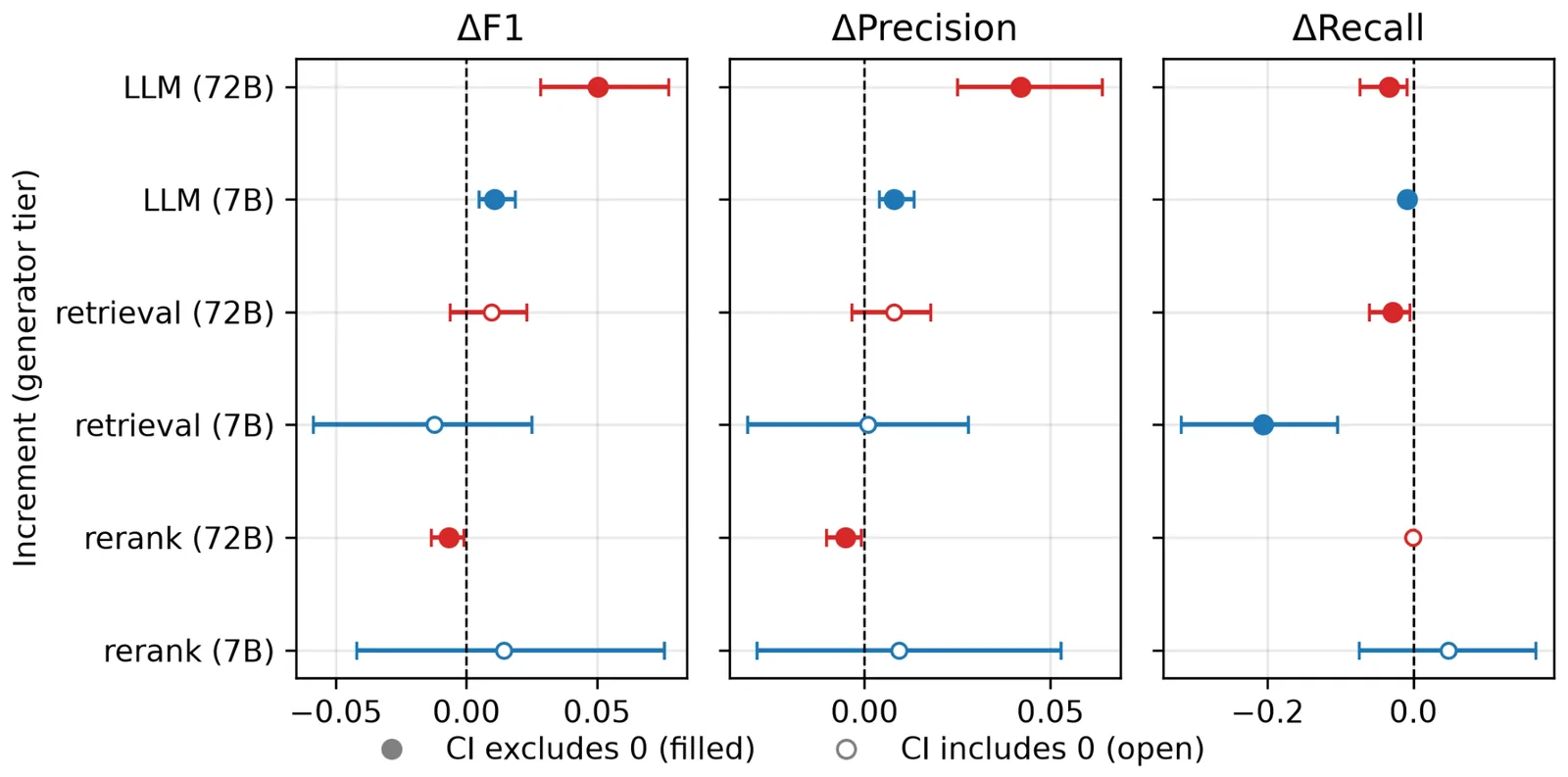
Forest plot of stage-wise increments (ΔF1/ΔPrecision/ΔRecall), n = 40, matched recall = 0.9. Filled = CI excludes 0. Color encodes the generator tier, the same information as in the row labels (red, 72B; blue, 7B); open circles are increments whose 95% CI includes zero. Horizontal bars are the 95% CIs and the dashed vertical line marks zero.

The retrieval increment (llm_retrieval − llm) is the primary preregistered quantity. For both generator sizes, the 95% CI for the F1 and precision increments includes zero (7B: F1 −0.012 [−0.059, 0.025], precision +0.001 [−0.032, 0.028]; 72B: F1 +0.010 [−0.006, 0.023], precision +0.008 [−0.003, 0.018]), triggering the preregistered Alt-2 decision rule and yielding an honest null on the primary contrast. This is a null in the sense that there is *no detectable net benefit*, not a demonstration that the true effect is exactly zero. Its scale is bounded: at n = 40, the design would detect a true 72B F1 gain of ≈0.021 at 80% power (two-sided α = 0.05; bootstrap SE 0.0074), and the 72B interval excludes true benefits larger than +0.023 F1, well below the LLM-stage gain of +0.050 (SQ1). The 7B configuration is considerably noisier (minimum detectable effect ≈ 0.060 F1). The stage-wise increments and their intervals are shown in Figure 1.

Per-stream reporting makes the null sharper rather than softer; the median retrieval increment on F1 is essentially zero at both generator sizes (+0.0001 and 0.0000), so the typical stream is not merely statistically indistinguishable from unchanged but literally unchanged.

**Figure 2:**
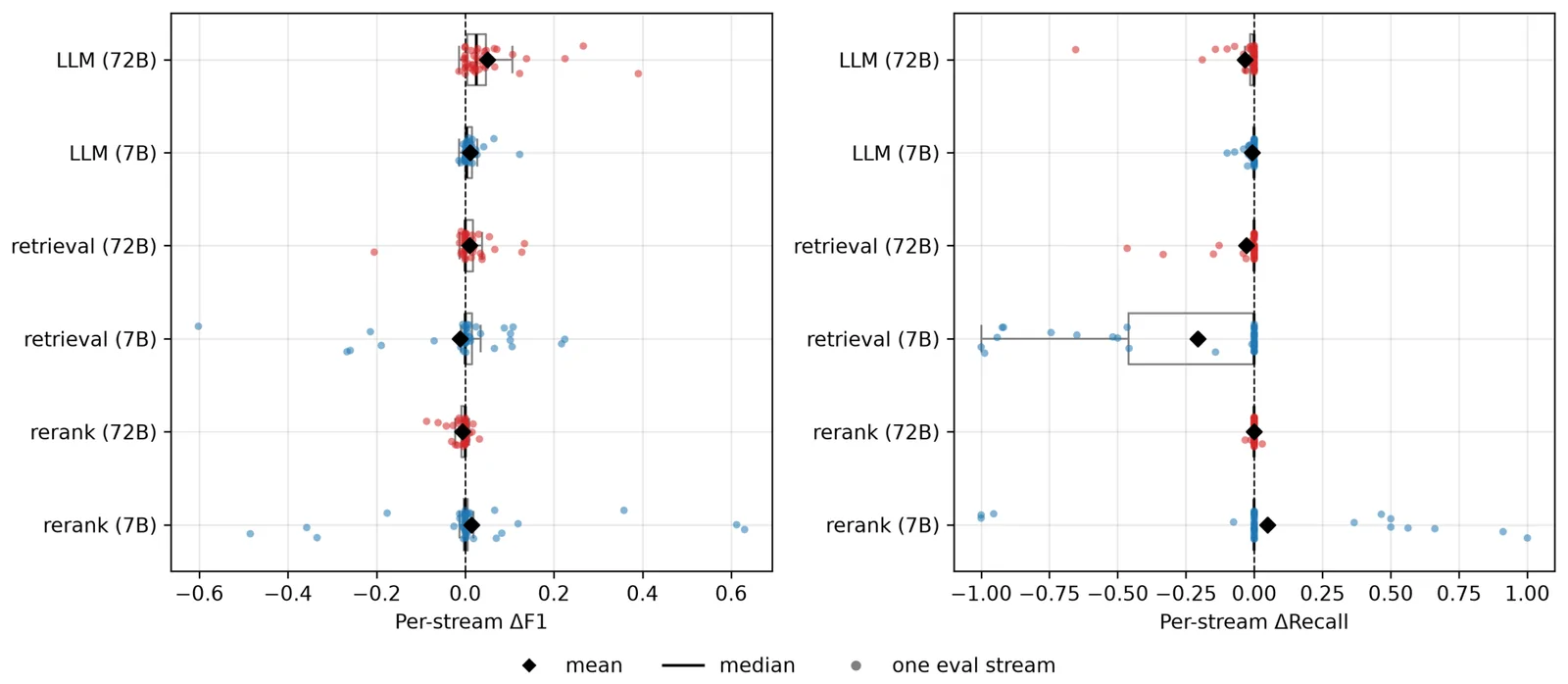
Per-stream distribution of each increment across the 40 evaluation streams for ΔF1 (**left**) and ΔRecall (**right**). Each point is one stream; boxes give the interquartile range, the vertical line the median and the diamond the mean. Point color encodes the generator tier, the same information as in the row labels (red, 72B; blue, 7B); the gray dot in the key is a neutral symbol and carries no tier meaning. Points are jittered vertically within each row so that overlapping streams remain visible, and the dashed vertical line marks zero. The retrieval recall increment at 7B illustrates why the mean alone is misleading: most streams are unaffected and a minority collapse.

Retrieval is nonetheless not inert. It significantly degrades recall at both generator sizes (7B −0.206 [−0.319, −0.104]; 72B −0.029 [−0.060, −0.005]), so adding retrieved historical context measurably changes triage behavior while delivering no detectable F1 or precision gain. The per-stream distributions in Figure 2 show the degradation is concentrated rather than pervasive, which changes the correct summary. At 7B, the median stream is unchanged, and 67.5% of streams show exactly zero change; the mean of −0.206 is produced by the remaining 32.5%, in which detection collapses, with several streams losing recall entirely. At 72B, 82.5% are unchanged and only seven streams move at all. Removing the five streams that drive the effect more than halves it at 7B (−0.206 to −0.100) and all but removes it at 72B (−0.029 to −0.001). We report the mean over all 40 streams, which is the preregistered quantity, and give the distribution alongside it. The accurate statement is not that retrieval costs 0.2 recall on average, but that it leaves most streams untouched and occasionally suppresses detection outright, a distinction a practitioner reading only the mean would miss.  

Where those affected streams come from matters for how far the result travels, so we stratify the increment by benchmark family. The recall degradation is carried almost entirely by the NAB series. On the 19 SKAB industrial process streams, it is indistinguishable from zero at both generator sizes (7B −0.048 [−0.145, +0.000]; 72B −0.0004 [−0.0011, +0.0000]), while, on the 21 NAB streams, it is large and significant (7B −0.349 [−0.518, −0.190]; 72B −0.055 [−0.112, −0.010]). We therefore do not claim that retrieval degrades recall on industrial process data, as, on the industrial subset of our own evaluation set, it does not. The preregistered primary contrast is null on both subsets separately as well as pooled, on both F1 and precision, so the headline null is not an artifact of mixing sensor families.

One further check cuts the other way and we report it too. Because the increments are zero-inflated, the interval on the mean and the consistency of direction are different questions. An exact sign test over the streams that actually move finds the 72B retrieval increment on F1 to be positive in 18 of 25 (*p* = 0.043) and the 7B increment to be positive in 20 of 29 (*p* = 0.061). The preregistered rule is the interval on the mean, which includes zero, and we do not overturn it. Nevertheless, the honest summary is that retrieval nudges F1 upwards in more streams than it harms while the average effect stays indistinguishable from zero, not that retrieval does nothing. The reranking increment is negative in only 13 of 19 moving streams (*p* = 0.167), a further reason prompting us to withdraw it.

### 4.4. SQ3: Reranking Increment

The reranking increment (llm_rerank − llm_retrieval) is not significant for the 7B generator on any detection metric (F1, precision, recall and PA-F1 CIs all include zero), although its faithfulness increment is a small significant positive (+0.008 [0.001, 0.014]; see SQ4). For the 72B generator, reranking produces a small but statistically significant decrease in F1 (−0.007 [−0.013, −0.001]), precision (−0.005 [−0.010, −0.001]) and PA-F1 (−0.007 [−0.014, −0.001]); the recall change is not significant (−0.0004 [−0.003, 0.002]). These decreases are small in magnitude (≈0.007 F1, arm-level 0.454 to 0.447) and survive Benjamini–Hochberg control of the false discovery rate across the 26 secondary increment tests (adjusted *p* = 0.036, 0.029, 0.038, respectively; Section 3.5). Table 1 shows the shape of this result: the 72B arm’s F1 rises at the LLM step and then flattens, while the 7B F1 barely moves.

The non-significant 7B recall increment shows the same pattern: its mean is positive (+0.048) but the median stream is exactly zero, and 70% of streams are unchanged and removing the five streams that drive it turns the mean negative (−0.049). Either sign is available depending on which streams are included.

We nonetheless do not draw a boundary condition claim from those 72B decreases because the effect does not reproduce. Section 4.7 re-estimates this increment under the nine configuration changes in which the reranking arm is run; the neighbor count and retriever axes vary only the retrieval stage and cannot speak to it. In none of the nine does the 72B penalty on F1 and precision remain distinguishable from zero. Three do return a significant negative effect, but on another metric or at the other generator size: PA-F1 at 72B under the 0.05 alarm rate (−0.0022 [−0.0042, −0.0007]), PA-F1 at 7B under the 0.10 rate (−0.0372 [−0.0873, −0.0005]) and precision at 7B at recall 0.5 (−0.0409 [−0.0831, −0.0049]). We report these rather than restricting the comparison to F1 after the fact, the practice this paper criticizes in its own primary contrast. What they show is a penalty that is real somewhere in the design space but unstable in metric, sign and generator size. One split leaves the point estimate essentially unchanged (−0.0065 against the frozen −0.0067) with a wider interval, consistent with an effect too small to resolve at n = 40. Elsewhere, the point estimate itself falls near zero, and, under the second generator family, reranking even raises recall significantly (+0.047), a direction absent from the frozen configuration. We therefore report the frozen result as observed but unreplicated and withdraw the claim made in the original submission that it establishes an in-domain boundary condition on the “reranking helps” prior.

### 4.5. SQ4: Explanation Faithfulness

**Figure 3:**
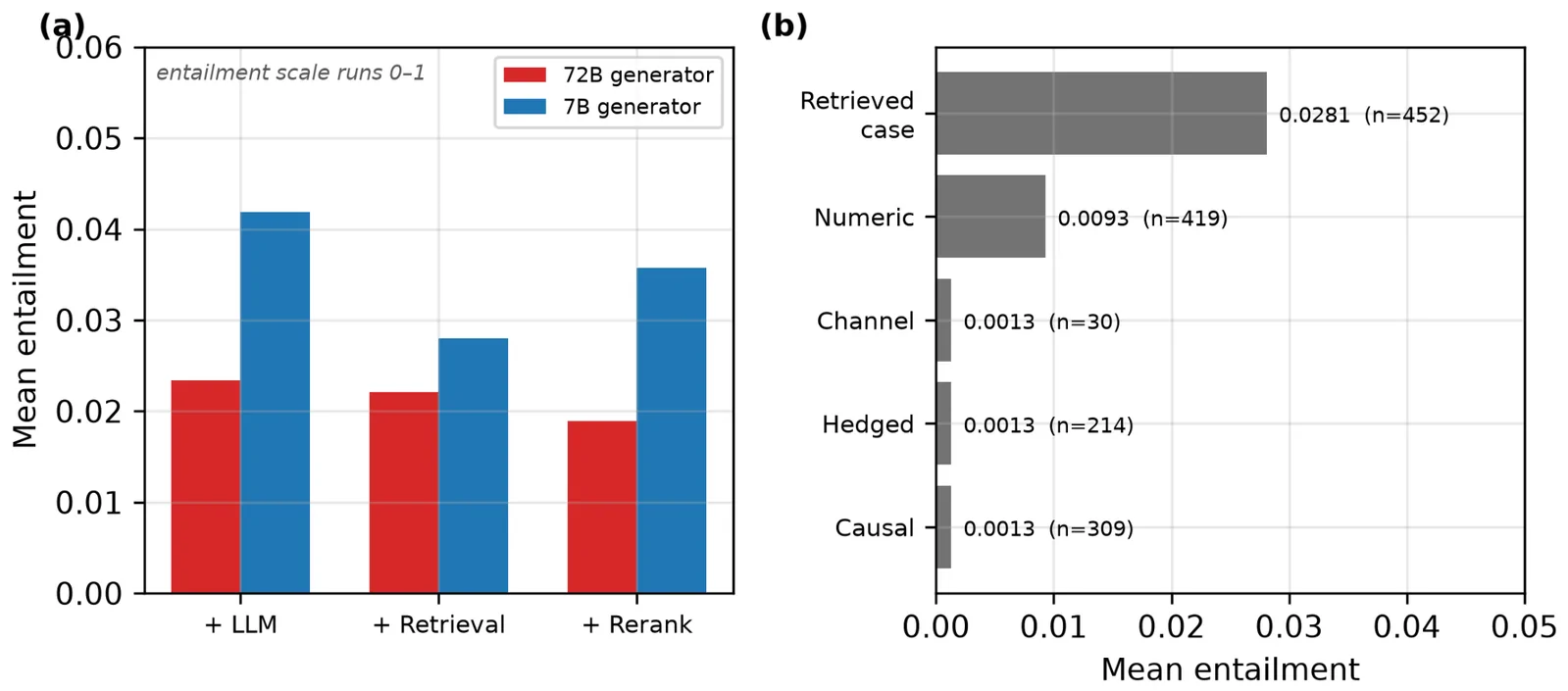
Local NLI explanation faithfulness. (**a**) Mean entailment by arm and generator size. The axis is expanded to show the differences, and all arms sit near the raw-entailment floor on a scale that runs to 1.0. (**b**) Mean entailment by claim type over the re-scored sample (8 streams, 664 explanations, 1467 claims) with the five claim types defined as references to a retrieved case, numeric statements about sigma levels or percentiles, causal attributions, hedged assertions and channel names; a residual “other” category (n = 43, 2.9%) is omitted. In panel (**a**), the LLM increment (both sizes) and the 7B rerank increment (+0.008) exclude zero; the retrieval increment does not, at either size (Table S0). Causal, hedged and channel claims sit indistinguishably at the floor (all 0.001), while numeric and retrieved-case claims score higher—the explanations are least grounded where they assert causes, which is the part an operator would act on.

Faithfulness is undefined for the detector-only arm, which produces no explanation, so the quantity we label the LLM increment is not a stage-to-stage contrast; it is the LLM arm’s absolute faithfulness measured against a floor of zero (7B 0.042 [0.019, 0.073]; 72B 0.023 [0.014, 0.034]). What it establishes is that generated explanations entail a small but non-zero share of their supporting context. It does not establish that a generation stage improves on a comparable baseline, because no such baseline exists here. The retrieval and reranking faithfulness increments below *are* stage contrasts, since both arms produce explanations. The reranking increment in faithfulness is also a small significant positive for the 7B generator (+0.008 [0.001, 0.014]). However, the retrieval increment in faithfulness includes zero for both generator sizes (7B −0.014 [−0.047, 0.011]; 72B −0.001 [−0.012, 0.010]), so retrieved historical context does not measurably increase how well explanations are entailed by their cited evidence. A calibration probe locates these values on the scorer’s own scale: of 36 hand-written claim/premise pairs of the study’s form (12 entailed, 12 contradicting, 12 neutral), entailed pairs score 0.9933 on average (minimum 0.9869), contradicted 0.0000 and neutral 0.0002. The scale is therefore not compressed in this domain, and the arm-level values of 0.019–0.042 (Table 1) are not an artifact of an instrument that never emits high probabilities. Figure 3 shows how narrow the band is.

To characterize where the low absolute values come from, we re-score a dedicated sample from the frozen configuration at the level of individual claims (8 streams, 664 explanations, 1467 claims), classifying each claim by what it asserts. The distribution is uneven in an interpretable way. Claims that reference a retrieved past case score highest (mean entailment 0.028, 31% of claims), which was expected because the premise for the retrieval configurations contains those case descriptions. The scorer does respond when a claim points at something the premise actually states. Numeric claims about sigma levels and percentiles score lower (0.009, 29%). Causal claims, which assert that the window indicates, suggests or is explained by something, score essentially zero (0.001, 21% of claims, none above an entailment of 0.5). Across all claim types, only 0.3% exceed 0.5. The low aggregate figure is therefore not uniform noise. The explanations are least grounded precisely where they assert causes, which is the part a human operator would be most inclined to act on.

To check the automated measure against human judgment, **three annotators independently label a blind sample** (premise and claim visible, score hidden) as *entailed*, *neutral* or *contradicted*. Judgment is at the level the scorer works at: the 60 sampled explanations are split by the scorer’s own sentence splitter into **133 claims**, so annotator and instrument read the same string. Agreement is moderate (Fleiss’ κ = 0.555; pair-wise Cohen’s κ 0.488–0.613), and majority consensus resolves all 133 items. The sample comes from the dedicated run above: 8 streams and the 2 explaining arms, unevenly split (33 no-retrieval, 27 retrieval). That limits what the counts can carry.

Two features bound what follows. The annotators differ in strictness more than in judgment. They call 25, 31 and 53 claims entailed. The strictest reader’s set is a subset of the most permissive reader’s, and all 31 disagreements between those two run one way. The number a reader calls grounded is therefore an operating point (25–53) rather than a point estimate. Furthermore, the descriptive/verdict partition below was made *after* the labels were collected, by a deterministic regular expression never shown to the annotators. The instruction sheet they did see named *“therefore a real anomaly”* as extrapolation to be labeled neutral. This overlaps the partition, so the verdict result is in part a confirmation that the rubric was applied consistently.

**Within that bound, the shortfall has a location.** Consensus judges 36 of 133 claims entailed, 96 neutral and 1 contradicted, and the two kinds of claim differ: Claims restating the premise’s measurements are often grounded (36 of 69). Claims delivering the system’s verdict are almost never grounded; consensus calls all 64 neutral, and the annotators individually call 64, 64 and 61 of 64 neutral. The most permissive reader allows 2 entailed there, against 51 of 69 descriptive claims. Two things keep this from resting on the instruction sheet: the sheet said nothing about descriptive claims, which is the side that varies, and the scorer reaches the same place without having seen it. Over the 1467 claims of the claim-type analysis, it puts causal claims at 0.0013 mean entailment, none of the 309 above 0.5, at the floor with hedged and channel claims and an order of magnitude below the numeric and retrieved-case types. A rubric and an instrument sharing no wording agree on which part of an explanation its evidence does not carry.

**The scorer scores absolutely low, and its ordering is validated only where the measure varies.** The level is unambiguous; its argmax label is *neutral* for **all 133 claims** and only 5 exceed 0.05, while the annotators call 25 to 53 entailed. The measure is therefore a relative index and not a probability of groundedness, which is how Section 4.2, Section 4.3 and Section 4.4 use it. Pooled over both arms, the ordering agrees with consensus (Spearman ρ = +0.38, n = 133), but the pooled correlation and the arm-level faithfulness comparison share a cause (premise content), so neither licenses the other, and we do not read the correlation as validating cross-arm comparison. Split by arm, it agrees within the retrieval arm (+0.29, n = 68) and not within the no-retrieval arm (−0.06, n = 65), where the scorer is nearly constant (mean 0.0019, SD 0.0038, no claim above 0.02) and there is no variance to order. Both correlations treat the 133 claims as independent, which they are not; they nest within 60 explanations and 8 streams, so we read them as directional rather than as tests.

### 4.6. SQ5: Generator Capacity (Exploratory)

This contrast is not statistically tested and is not a preregistered headline claim (n = 40 per generator size). Compared descriptively, the 72B generator shows a larger LLM increment in F1 (+0.050 vs. +0.011), a sign flip in the retrieval increment (from a negative point estimate at 7B to a positive one at 72B, both non-significant on F1/precision) and markedly smaller recall suppression under retrieval (−0.029 vs. −0.206). These are consistent, in-domain observations that do not by themselves establish a capacity-dependent mechanism.

The second generator family added at revision (Section 4.7) shows that the last of these observations is not a capacity effect at all: Llama-3.1-8B, comparable in size to Qwen2.5-7B, shows no retrieval-induced recall loss whatever (+0.018 [−0.008, 0.049]) where Qwen2.5-7B loses 0.206. The suppression is therefore specific to that generator rather than a property of small generators, and we revise our reading accordingly: what the original contrast recorded as a capacity effect is, at least in part, a model effect. The LLM increment, by contrast, does behave consistently across families—it is significant in both Llama sizes, and larger at 8B (+0.044) than the corresponding Qwen 7B value (+0.011).

### 4.7. Sensitivity of the Findings to Design Choices

The results above come from one configuration. To test whether they depend on it, we re-run the audit under the seven sensitivity axes and thirteen configurations set out in Section 3.7, varying one factor at a time against the frozen baseline. Every axis is run over the full 40-stream evaluation set with the same bootstrap procedure. Table 3 summarizes the outcome for the preregistered primary contrast and the two effects that accompany it; per-axis detail is in the Supplementary Material.

First, the preregistered null is robust. On the criterion stated in Table 3, it holds in seven of the eight configurations that change the pipeline’s design (six of seven if the low-resolution z-score configuration discussed below is set aside), spanning both generator families, three splits, three neighbor counts, two retrievers and two detectors. One design configuration breaks it, in the opposite direction to everything else: at k = 10 the 7B precision increment is significantly positive (+0.026 [0.003, 0.052]) while its F1 increment stays null. Under the second generator family, the 70B increment is also null (+0.0092 [−0.0137, +0.0379]), overlapping the frozen 72B interval.

Not all of these nulls carry the same weight, and it would be misleading to count them as if they did. A null is informative only when its interval is narrow enough to exclude an effect that would have mattered. The natural yardstick is the LLM increment on the same metric, generator and candidate set. Where the candidate set is regenerated or the generator family changes (C-1 and C-4 to C-8, Section 3.7), that is each configuration’s own value, not the frozen +0.0502, from which it can differ six-fold (+0.0081 at alarm rate 0.10). Where only the retrieval stage varies and the arms below it are reused (C-2 and C-3), the frozen value is the one that applies. Using that yardstick, 15 of the 42 configuration-level nulls exclude an effect that large (*bounded*) and 27 do not (*undetected*; Table S17), with every moved operating point null falling into the latter group. The strong reading of the null, that retrieval adds materially less than the generator stage, is supported at 72B and unsupported at 7B. Here, the honest statement is that this study lacked the resolution to test it.

Second, the null is specific to the oracle operating point, and we test three label-free alternatives rather than one so that this does not rest on a single arbitrary threshold. Setting the candidate threshold at a fixed alarm rate of 0.02, 0.05 or 0.10, using no labels at any point, turns the retrieval increment on F1 into a significant *loss* at the 7B size in all three (−0.039, −0.038, −0.039). The three values are so close that the effect is evidently not an artifact of where the budget is placed. At 72B, the loss is significant at 0.02 and 0.05 (−0.015, −0.018) and marginally null at 0.10 (−0.015 [−0.035, +0.002]). The retrieval increment on precision, the other preregistered primary metric, is null at every rate and both sizes, so, at these rates, retrieval forfeits detection without buying precision in exchange.

The LLM stage behaves differently, and, here, the earlier framing in this paper is too strong. Its F1 increment does not simply vanish once labels are removed; it is null at 0.02 and 0.05 but significantly positive again at 0.10 (+0.008 [0.003, 0.015]), about a sixth of the +0.050 it delivers under the oracle threshold. Its precision increment survives more readily still, remaining significant at 0.05 and 0.10 (+0.026, +0.034). What the operating point governs is therefore the size of the LLM gain rather than its existence.

Table 4 relates the LLM gain to one property of the candidate set the detector hands over: how much recall it already contains. It reports two families that must not be read as a single curve: the preregistered fixed-recall grid, which holds the thresholding policy at the oracle, and the label-free rates, which change the policy itself (Section 3.7). Candidate recall and candidate precision remain mechanically inverse within the grid, however, so recall is separated from the policy, not from precision.

Within the fixed-recall grid, the gain falls across the three points (Table 4), becoming null at candidate recall 0.622. The 72B LLM increment on precision over the same three points is +0.042 [0.025, 0.064], +0.030 [0.017, 0.046] and +0.039 [0.011, 0.085]—significant at all three and not ordered by recall, which is what Section 5.1 takes up. The ordering is a property of F1 under a fixed policy; on the same three points, the point-adjusted variant is nearly flat at 72B (+0.056, +0.055, +0.042) and is not ordered at all at 7B (+0.013, +0.014, +0.008), so the bound below is stated for F1 and not for segment-credited scoring. The first two F1 increments are individually significant and strictly ordered, so the ordering between them is interpretable. Their intervals nonetheless overlap, and we do not test the pair-wise differences, so the ordering is read as a bound on how far the gain can persist rather than as a dose–response curve.

One rival explanation is excluded by the design and one is not. Candidate purity is not excluded, and we say so rather than claiming otherwise. Candidate precision *rises* across exactly these points (0.269, 0.321, 0.382), and, for a stage whose only action is suppression, a purer candidate set leaves less to remove. A headroom account therefore predicts the same falling gain that a recall account does; the two are not separated here. The number of candidates is excluded; it nearly doubles from 896 to 1785 events between the first two points while the gain more than halves. What changes together with the gain is the recall the candidate set already contains.

The grid also breaks the preregistered null. At both lower recall targets, the retrieval increment on precision is significantly positive at both generator sizes (at recall 0.7, +0.015 [0.001, 0.030] at 72B and +0.039 [0.009, 0.071] at 7B; at recall 0.5, +0.031 [0.005, 0.066] and +0.064 [0.011, 0.126]). The F1 increment stays null and the recall increment remains significantly negative. Several stratified recall intervals in Table S15 have an endpoint at exactly zero rather than rounded to it. The per-stream increments are zero-inflated, so the bootstrap distribution carries an atom at zero and the percentile endpoint lands on it. Those verdicts are one-sided bounds, not two-sided nulls. Retrieval therefore trades recall for precision, and the trade becomes statistically visible once the candidate set is tightened. The two movements cancel in F1, which is why the primary contrast reads null at the frozen operating point. What we withdraw is not the null itself, which is defined at the preregistered operating point, but any reading of it as evidence that retrieval does nothing. On these two preregistered points, it does something specific and measurable: it shifts the operating point rather than improving the trade-off.

The label-free family is consistent with this but cannot establish it, and we do not claim otherwise. There, candidate recall and precision move together, and its two lowest settings are both null, so their ordering carries no information (−0.004 at recall 0.069 against −0.005 at 0.154). A further property varies that we cannot separate: a fixed-rate threshold flags a fixed share of timepoints while the oracle flags whatever reaching a recall target requires, so one suppression decision covers different numbers of timepoints. This confound is the reason the fixed-recall grid was preregistered as a separate test.

Stratified by benchmark family, the industrial reading is simpler than the pooled one, and, for this journal’s readers, it is the more relevant cut. On the 19 SKAB industrial process streams, the LLM increment is significant only at the oracle target (+0.020 [0.013, 0.027]). It is null at both lower targets of the grid, null at every label-free rate, and, at rates 0.02 and 0.10, **exactly zero on all 19 streams**. The generator confirms every candidate it is given, so the arm’s output is identical to the detector’s. The +0.008 the pooled figure records at rate 0.10 comes entirely from the 21 NAB streams (+0.015 there). At the operating points a deployment can actually reach, this triage stage does nothing measurable on industrial process data, and the gain visible under the oracle threshold is a property of a candidate set selected with labels.

At the tightest budget, the stage reaches its limit outright: at rate 0.02 the 7B generator confirms every candidate and suppresses none, making it byte-for-byte identical to the detector it was meant to improve at about three seconds per alarm, and the larger generator behaves the same way on the industrial subset at about thirty seconds. Why the ordering takes this form is a structural property of post-processing rather than an accident of this pipeline and we take it up in Section 5.1.

Third, one effect we previously read as a size effect is a model effect. The retrieval-induced recall loss at 7B (−0.206) does not appear in Llama-3.1-8B (+0.018), and it is present at k = 1 as strongly as at k = 5 while halving at k = 10, the opposite of what a long-context burden would predict. We accordingly no longer align this observation with the small-model retrieval utilization literature (Section 5).

Fourth, the retrieval increment in faithfulness is null in 21 of the 24 configuration-by-generator-size cells of Tables S1–S12, and the exceptions run both ways: they are significantly negative at 7B under k = 1 (−0.0282 [−0.0603, −0.0034]; Table S4) and under the rolling z-score detector (−0.0490 [−0.0995, −0.0055]; Table S7) and significantly positive for Llama-3.3-70B (+0.0226 [0.0076, 0.0404]; Table S8).

One axis carries less evidence than the others. Matching recall at 0.9 with the rolling z-score detector requires a much lower threshold, so flagged points merge into long candidate events. The median stream carries one candidate event under that detector against 13 under the frozen one, and between 72% and 93% of streams show an increment of exactly zero. A null in that regime reflects absence of resolution as much as absence of an effect, so we do not read the z-score column as independent confirmation. Matching an operating point does not by itself match the conditions under which a comparison is made.

The stream-level bootstrap treats the 40 evaluation streams as independent, but they arrive in six benchmark families (Section 3.1), the 19 SKAB streams among them forming a single testbed, and positive within-family correlation would make the intervals too narrow. Resampling those six families rather than the streams (B = 10,000, seed 42) changes no point estimate and only widens intervals. The frozen point null and the recall degradation stand as reported. A wider interval cannot overturn a result that already includes zero (72B F1 +0.0095 [−0.0073, +0.0570]), and the 7B recall loss stays significantly negative (−0.2063 [−0.4960, −0.0566]). The grid’s precision break does not come through intact: two of its four cells still exclude zero and two do not, and the survivors do not line up. At recall 0.7, the 7B increment survives (+0.0388 [+0.0049, +0.1110]) and the 72B one does not (+0.0148 [−0.0008, +0.0687]); at recall 0.5, it is the other way round (72B +0.0310 [+0.0053, +0.0729]; 7B +0.0639 [−0.0020, +0.1456]). Neither the recall target nor the generator size orders which cells survive, so the family-level result weakens the break rather than locating a condition under which it holds. The preregistered analysis is the stream-level one. This is a check on the independence it assumes, and we report what the check returns.

### 4.8. Computational Cost

Streaming triage is latency-sensitive, so we measure the per-alarm cost of each stage on the hardware used for the audit through the same code paths and serving configuration as the main experiment (one DGX Spark GB10 node; llama.cpp, Q8_0, temperature 0). Table 5 reports the result.

The generator dominates by three orders of magnitude: a 72B triage decision costs roughly 33 s of accelerator time per candidate alarm, against 15 ms for the retrieval it is conditioned on. Even the 7B generator, at about 3 s per alarm, is some 200 times the cost of the retrieval stage. One comparison has to be made before this cost is read as buying anything. Within the same oracle policy, moving the threshold from recall 0.9 to 0.5 raises the detector’s own F1 from 0.394 to 0.444 (Table 4), which is what the 72B generator delivers at the frozen point for about thirty seconds of accelerator time per alarm. The generator is therefore not buying an F1 level that a threshold could not reach; what it buys is that level while candidate recall stays at 0.978, where the grid move gives up recall down to 0.622. The cost profile reinforces the audit’s conclusion rather than qualifying it. The expensive stage is the one whose benefit disappears once the operating point is set without labels, and the cheap stage is the one that never improves F1 at any setting, buying precision only by giving up recall.

### 4.9. Detector Baseline (Threshold-Independent)

**Figure 4:**
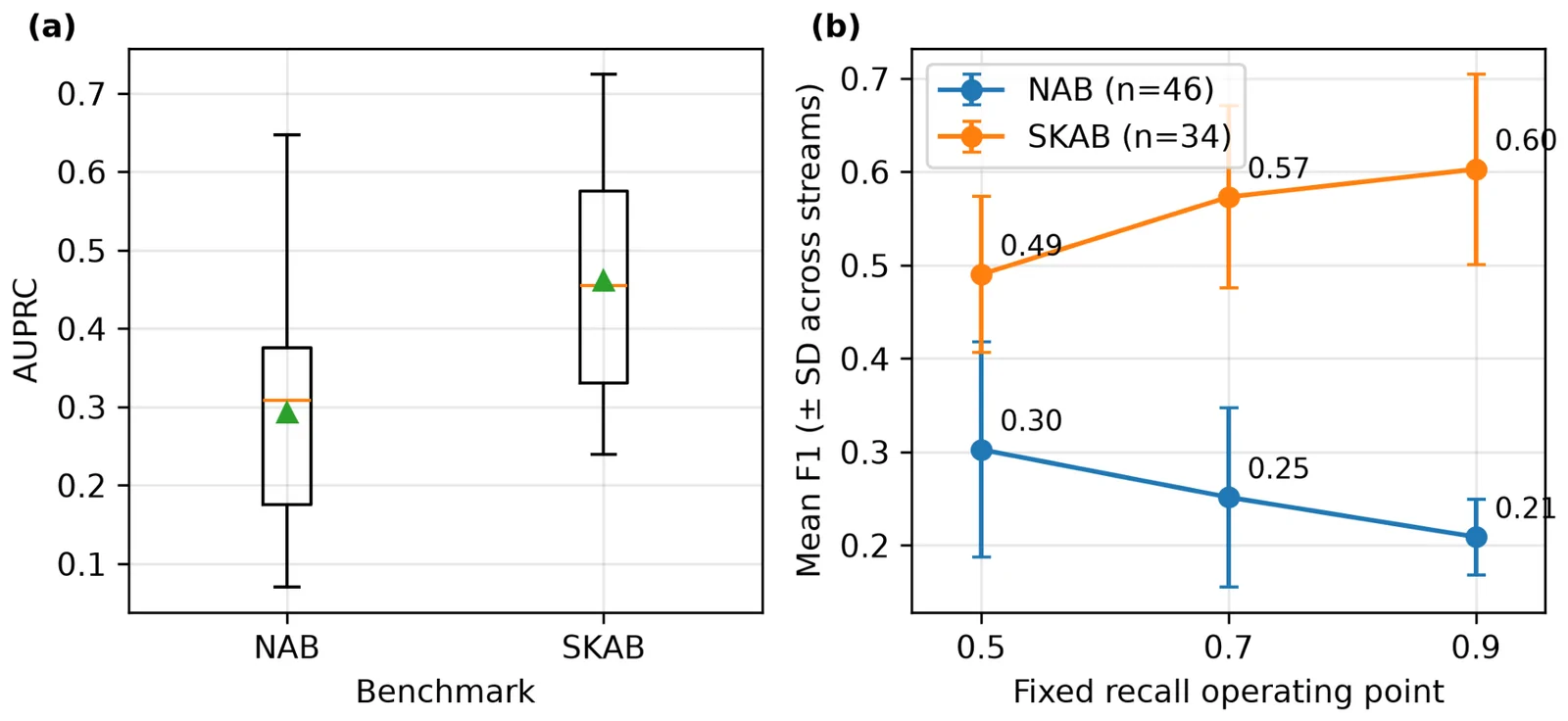
Threshold-independent detector baseline across all 80 streams. (**a**) Distribution of per-stream AUPRC by benchmark. Boxes show the interquartile range, the orange line the median and the green triangle the mean. (**b**) Mean per-stream F1 at each fixed-recall operating point, with error bars giving the standard deviation across streams.

Across the pooled 80-series baseline set, AUPRC averages approximately 0.29 for the 46 NAB real-category series and 0.46 for the 34 SKAB series, and, at recall = 0.9, mean F1 is approximately 0.21 for the NAB and 0.60 for the SKAB. PA-F1 is at least as high as the raw F1 at every grid point (0.23 against 0.21 for the NAB, 0.65 against 0.60 for the SKAB), which illustrates the inflation risk of the point adjustment protocol. Figure 4 shows the gap: the SKAB’s AUPRC distribution is shifted upward but overlaps the NAB’s substantially (medians 0.46 against 0.31), and, as the recall target rises, the NAB’s mean F1 falls while the SKAB’s rises. The arm-level operating point is fixed independently on the 40-series eval subset (Section 3) and is not derived from this pooled grid.

## 5. Discussion

### 5.1. What Bounds a Confirm-or-Suppress Stage

A stage that only confirms or suppresses can remove candidates but never recover a missed one, so it can trade recall for precision and nothing else. The precision it buys is roughly invariant across the grid, with the 72B precision increment being significant at all three recall targets and not ordered by recall (Section 4.7). What changes is the exchange rate at which precision is bought: at the frozen point, a unit of precision is worth 1.23 units of F1 against 0.093 for a unit of recall; by candidate recall 0.622, these are 0.768 and 0.290. The cost of one mistaken suppression grows 3.1-fold while its benefit does not, and that is what drives the F1 gain down. The oracle threshold flatters this stage precisely because the oracle is *defined* by recall. Fixing recall at 0.9 creates the headroom the gain needs, and candidate count moves the wrong way to explain it, while candidate purity does not. The two accounts are not separated here (Section 4.7). Because those two partial derivatives behave this way for any precision and recall, the grid separates the recall target under a fixed policy but not that account.

The following is stated as a bound observed across this pipeline’s fixed-recall grid (0.9, 0.7, 0.5) rather than a finding: the F1 gain a confirm-or-suppress post-processor can add is capped by the operating point of the candidate set it is handed, i.e., the exchange rate between precision and recall at that point. Its precision gain is not, and rises as the candidate set tightens. Evaluating such a stage at an operating point chosen with labels therefore reports a ceiling rather than a deployment estimate. That is why a matched operating point is not by itself sufficient; the point must also be one the deployment can reach. The bound rests on the preregistered fixed-recall grid rather than on a confounded comparison, since only there is recall isolated (Section 4.7). It remains an addition made at revision, reported without multiplicity correction, and what it establishes is that the ordering survives when the rival explanations are held fixed, not that the relationship is a fitted dose–response curve.

### 5.2. Interpreting the Increments

The four-arm decomposition separates what “RAG-LLM post-processing” buys into three distinguishable components, and the LLM increment, though real and substantial in precision terms at 72B, is not a general improvement; it trades recall for precision (SQ1). One comparison is direct: the nearest prior system shares a benchmark with us. RAAD-LLM was validated on a plastics manufacturing plant **and on the SKAB** [6], the industrial half of our evaluation set, and reported large gains. On our 19 SKAB streams, the retrieval increment is null (+0.002 [−0.002, +0.008] at 72B). The published account cannot adjudicate the difference. Its headline 70.7% to 88.6% comparison is against the authors’ own earlier model on the plastics dataset; the SKAB result is reported separately, and their Table II does pair a retrieval-free model with the retrieval one there (F1 0.56 against 0.74). That pair cannot be read as a retrieval increment; the two arms sit at different recall (0.68 against 0.89) and differ in more than the retrieval stage. Neither comparison isolates an LLM increment from a retrieval increment at a common operating point, which is the measurement gap this study is about. Three explanations remain open—a different task definition, a different detector and candidate set or the absence of a matched operating point—and we cannot separate them. The same holds for EagerLog’s double-digit F1 improvement from active learning-augmented log retrieval [5], whose reported gain is a composite system figure (Table S19). Our own decomposition places the gain in the LLM stage.

The retrieval increment, the preregistered primary contrast, is the honest null this study was designed to expose (Results, SQ2): a *bounded* null at 72B rather than a claim of exact zero, since the interval excludes any gain approaching the LLM stage’s, and an *undetected* one at 7B, where the design lacks the resolution to make that comparison. Retrieval is not inert; at the frozen point, it significantly degrades recall without buying precision, and, at the two lower preregistered targets, it buys precision while still costing recall (Section 4.7). What it does not do at any of them is add decision-relevant information beyond what the LLM stage already extracts from the window description.

Both the lost-in-the-middle observation [37] and the small-model retrieval utilization failures reported under oracle retrieval (Section 2.4) [39] describe models struggling to exploit retrieved context. On the frozen configuration alone, that appeared to echo the recall degradation we observed at 7B. The sensitivity analyses withdraw that alignment; a second model of comparable size shows no retrieval-induced recall loss, and the loss shrinks rather than grows with the amount of retrieved context, the opposite of what a long-context burden predicts (Section 4.7). We therefore report the degradation as specific to the generator we audited rather than as in-domain support for the utilization bottleneck literature. That the alignment looks convincing for one configuration and dissolves under two independent checks is itself an instance of the paper’s argument.

The reranking increment produces a small, statistically significant degradation in F1 and precision with the 72B generator in the frozen configuration, surviving false discovery rate correction across the secondary increment family (Results, SQ3). It does not, however, reproduce. In none of the nine configuration changes in which that arm is run does a penalty on F1 or precision remain distinguishable from zero at 72B, though three return one on another metric or at the other generator size (Results, Section 4.7). We therefore treat it as observed but unreplicated. That reranking improves generation is a well-documented prior (Section 2.5), and, on the one contrast that matches ours, it is not silent. The nearest study with a no-reranking baseline reported rerank-only as its preferred default, with the best accuracy–latency balance [46]. Our arm sits in that configuration, so the frozen observation runs against the prior rather than extending its cascading caution—a further reason to treat an unreplicated effect as unreplicated. What survives is narrower: in streaming triage, the reranking increment has no stable sign, so the prior does not transfer.

Explanation faithfulness sits near the raw-entailment floor across all LLM-based arms and is interpreted only comparatively. Three independent annotators tighten that restriction rather than relax it. They judge 25 to 53 of 133 claims as entailed where the scorer judges none, so no absolute reading is available. Their ordering agrees with the scorer’s only across arms (ρ = +0.38 pooled, +0.29 and −0.06 within arms), so the claim-level ordering is not validated either (Results, SQ4). The retrieval increment, which, unlike the LLM arm’s value, *is* a step between two explaining arms, includes zero at both generator sizes. Retrieved historical context does not make explanations more entailed by their cited evidence. This echoes two faithfulness audits from adjacent domains: up to 57% of citations in RAG outputs fail a faithfulness criterion, reflecting post hoc rationalization rather than reliance on the cited evidence [32], and LLM-generated explanations of personal sensing data overreach their evidence [36]. These works operationalize faithfulness differently from our claim-level NLI entailment but converge on the same concern, which our result extends to industrial streaming sensor anomaly explanation: retrieval augmentation does not, by itself, close the faithfulness gap.

### 5.3. Methodological Contribution

None of these findings is separable from a single composite accuracy comparison, and none is trustworthy unless the pipeline being measured is actually functional. The most transferable part of that second contribution is the preregistered build integrity gate (Section 4.1). This is not a formality; an earlier instance of this same pipeline ran retrieval as fixed boilerplate and never invoked the generator, a failure invisible to an aggregate accuracy score. Verification itself is not new; surveys of retrieval-augmented generation evaluation already distinguish component-level assessment [30]. What we add is that the gate is preregistered, executed before any increment is computed and released as a runnable suite bound to a specific decomposition. The companion determinism gate ships with an injection control confirming such a check is able to fail (Section 3.7). Others can apply it rather than accept a prose assurance.

### 5.4. Limitations

Stochastic components are fixed to a single seed: the detector is fitted with seed 42 and generation is greedy at temperature 0, so decoding is deterministic but detector fitting is not repeated across seeds. The corpus/eval split is varied at two further seeds (Section 4.7) and the primary null held, which bounds but does not remove this exposure. The bootstrap also resamples streams, which the six benchmark families of Section 3.1 make an optimistic unit; resampling families instead leaves the primary null and the recall loss in place and costs the grid’s precision break two of its four cells (Section 4.7). Results are drawn from a single case study of 40 streams across two benchmarks (NAB, SKAB) and do not support generalization claims; a different corpus, embedding model, detector, or prompt design could yield different increments. The templates used here are reproduced verbatim in Appendix A. The matched operating point equalizes the candidate set, not the final arm recall (7B retrieval recall falls to 0.763). Because the candidate threshold is calibrated per stream from ground-truth labels, the absolute precision and F1 levels upper bound what a deployed detector without label access could achieve. The increments are affected too; without the oracle, the retrieval increment turns into a significant loss and the LLM increment shrinks to a sixth of its value or below (Section 4.7).

Faithfulness is scored by a claim-level NLI protocol whose endpoints the calibration probe anchors but whose interior it does not, so we read it comparatively rather than as an absolute probability of groundedness. The probe was constructed by an author and establishes internal consistency only. Three independent annotators labeled the agreement sample at claim level (Fleiss’ κ = 0.555), so its verdicts are not an author’s self-audit. That check bounds the measure in both directions (Section 4.5). The level is systematically low and the claim-level ordering holds only where the measure varies (+0.29 in the retrieval arm, −0.06 in the near-constant no-retrieval arm). Because a premise carrying retrieved case descriptions lets any claim referring to them collect lexical credit, the arm-level faithfulness comparison is confounded with a change in the premise itself, as is the pooled scorer–consensus correlation reported above, and a premise-independent measure of grounding would be the better instrument. The eight-stream sample cannot size that effect, and we adjust no reported increment from it. This validation was added at revision and is covered by neither the freeze nor the amendment, and every analysis choice in it was made after the labels were collected, with no multiplicity control.

The capacity contrast (SQ5) is exploratory and unpowered at n = 40 per generator size; both sizes use identical Q8_0 quantization, prompts and decoding (Appendix A), so it varies nominal capacity without being a controlled mechanism test.

## 6. Conclusions

This study audited a constructed, build-verified RAG-LLM post-processing pipeline for streaming industrial anomaly triage by decomposing it into stage-wise increments at a matched operating point then re-running every component across seven sensitivity axes at revision. One distinction governs the reading: the preregistered result is the retrieval increment at the frozen operating point of recall 0.9, and the design axes added at revision are exploratory, carry no multiplicity control and cannot displace it. That increment is an honest null on both primary metrics, and it costs recall. It stays null under every change of detector, retriever, split and generator family tested. It is not evidence that retrieval does nothing.

At both points of the fixed-recall grid, preregistered in the original freeze, retrieval significantly improves precision while still costing recall, breaking the preregistered null on that metric at both targets. At the label-free points, where the candidate set is tighter still, it does not. One exploratory configuration also breaks it, at k = 10 and at 7B only, which is the number of exceedances that chance predicts across the twenty-eight uncorrected intervals of the design axes (seven configurations × two primary metrics × two generator sizes). On the nineteen SKAB streams, the industrial half of the evaluation set, the retrieval increment is null at the frozen operating point (Table S15). The grid’s precision break was not recomputed for that subset. The subset does show that the LLM gain there is significant only at the oracle target and null at every label-free rate.

What retrieval moves is the operating point, not the trade-off. The LLM stage delivers a detectable precision-weighted gain in both generator families, but its F1 increment shrinks with the detector’s own recall across the preregistered grid (Table 4). This is consistent with the bound derived in Section 5.1. An operating point chosen with labels therefore reports a ceiling, not a deployment estimate. The 72B reranking penalty reproduces in none of the nine configurations in which that arm ran (Section 4.7). We withdraw it, rather than narrowing it to the surviving metric. Retrieval does not improve explanation faithfulness in the frozen configuration, and, across the sensitivity axes, the increment is significantly positive in one configuration and significantly negative in two (Section 4.7). The consensus of three annotators found none of 64 verdict claims carried by the evidence.

The implication is methodological rather than architectural: a composite pipeline-level comparison would have reported these four components as a single undifferentiated “RAG-LLM improves detection” result, the framing this paper set out to interrogate. Separated at a common operating point, retrieval and reranking, both routinely assumed to help in the broader retrieval-augmented generation literature, do not transfer to this streaming triage setting without qualification. Each component’s contribution needs to be measured on its own, at an operating point the deployment can reach, rather than inferred from an aggregate score or from priors established on unrelated tasks such as open-domain QA.

These conclusions are drawn from a single case study on two benchmarks, one embedding/reranker configuration and one confirmatory matched operating point with a preregistered fixed-recall grid and three label-free rates around it. They establish that these effects occur here, not that they generalize. Applying the same decomposition protocol across a wider range of benchmarks, operating points, retrieval corpora and prompt designs would show how often the retrieval null recurs and whether the reranking sign stabilizes, and how far both are artifacts of this configuration. The other open task is grounding; the absolute faithfulness scale needs calibrating, and retrieval did not improve grounding, least of all in the verdicts.

## Figures and Tables

**Table 1:** Arm-level performance at the matched recall = 0.9 operating point by generator size as mean ± SD over the 40 evaluation streams. Arms are listed in the order they were added to the pipeline. Each arm is cumulative, so “+Rerank” denotes the full detector–LLM–retrieval–rerank pipeline. The SD is the spread across streams, not an uncertainty on the mean. Inference for every stage contrast is by stream-level bootstrap and is reported in Table 2. Faithfulness is undefined for the detector-only arm, which produces no explanation, and is shown as n/a rather than zero. Abbreviations: LLM, large language model; PA-F1, point-adjusted F1; SD, standard deviation.

Generator	Arm	Precision	Recall	F1	PA-F1	Faithfulness
72B	Detector only	0.269 ± 0.174	0.978 ± 0.024	0.394 ± 0.206	0.400 ± 0.207	n/a
72B	+LLM	0.311 ± 0.172	0.945 ± 0.110	0.444 ± 0.198	0.456 ± 0.196	0.023 ± 0.034
72B	+Retrieval	0.319 ± 0.169	0.916 ± 0.174	0.454 ± 0.196	0.469 ± 0.193	0.022 ± 0.024
72B	+Rerank	0.314 ± 0.170	0.915 ± 0.174	0.447 ± 0.198	0.462 ± 0.195	0.019 ± 0.018
7B	Detector only	0.269 ± 0.174	0.978 ± 0.024	0.394 ± 0.206	0.400 ± 0.207	n/a
7B	+LLM	0.277 ± 0.171	0.970 ± 0.036	0.405 ± 0.201	0.413 ± 0.200	0.042 ± 0.088
7B	+Retrieval	0.278 ± 0.180	0.763 ± 0.357	0.393 ± 0.229	0.424 ± 0.225	0.028 ± 0.021
7B	+Rerank	0.287 ± 0.191	0.812 ± 0.329	0.407 ± 0.237	0.421 ± 0.236	0.036 ± 0.019

**Table 2:** Stage-wise increments with stream-level bootstrap 95% confidence intervals (B = 10,000, seed 42) by generator size. Each increment is the difference between an arm and the one below it in the ladder, taken at the same matched operating point over the same 40 evaluation streams. Intervals that exclude zero are marked in the final column. The preregistered primary contrast is the retrieval increment on F1 and precision. PA-F1 and faithfulness increments are reported in Supplementary Table S0.

Generator	Increment	Metric	n	Mean Δ	95% CI	Excludes 0
72B	+LLM	F1	40	+0.050	[+0.028, +0.078]	yes
72B	+LLM	Precision	40	+0.042	[+0.025, +0.064]	yes
72B	+LLM	Recall	40	−0.033	[−0.073, −0.008]	yes
72B	+Retrieval	F1	40	+0.010	[−0.006, +0.023]	no
72B	+Retrieval	Precision	40	+0.008	[−0.003, +0.018]	no
72B	+Retrieval	Recall	40	−0.029	[−0.060, −0.005]	yes
72B	+Rerank	F1	40	−0.007	[−0.013, −0.001]	yes
72B	+Rerank	Precision	40	−0.005	[−0.010, −0.001]	yes
72B	+Rerank	Recall	40	−0.0004	[−0.003, +0.002]	no
7B	+LLM	F1	40	+0.011	[+0.005, +0.019]	yes
7B	+LLM	Precision	40	+0.008	[+0.004, +0.013]	yes
7B	+LLM	Recall	40	−0.008	[−0.015, −0.003]	yes
7B	+Retrieval	F1	40	−0.012	[−0.059, +0.025]	no
7B	+Retrieval	Precision	40	+0.001	[−0.032, +0.028]	no
7B	+Retrieval	Recall	40	−0.206	[−0.319, −0.104]	yes
7B	+Rerank	F1	40	+0.014	[−0.042, +0.076]	no
7B	+Rerank	Precision	40	+0.009	[−0.029, +0.053]	no
7B	+Rerank	Recall	40	+0.048	[−0.074, +0.169]	no

**Table 3:** Sensitivity of the main findings across the axes examined at revision. Both primary metrics are shown because the preregistration defines the contrast on precision *or* F1. A configuration preserves the null only when every interval, on both metrics and both generator sizes, includes zero, the stricter of the two readings the freeze admits, adopted and justified in Section 3.6. Each cell gives the direction of the increment and whether its 95% stream-level bootstrap CI excludes zero (*null* = includes zero). A cell names a generator size only where the two differ; an em dash marks an axis that leaves the arms entering that increment unchanged, so the frozen estimate applies without recomputation; **bold** marks a conclusion differing from the frozen configuration. The Configurations column sums to 13: 8 configurations that change the pipeline’s **design** (the denominator for the robustness count in the text) and 5 that change only the **operating point** at which the arms are compared. The design axes were added at revision and are exploratory. The two fixed-recall targets are not; they were preregistered in the original freeze and left unexecuted until revision, and they break the preregistered null on precision at both targets. Intervals carry no multiplicity correction across axes.

Axis	Configurations	Retrieval on F1 (Primary)	Retrieval on Precision (Primary)	Retrieval Effect on Recall	LLM Increment on F1
Frozen configuration	1	null	null	negative	positive
Neighbor count k	2 added (1, 10)	null in all 2	**null at k = 1, positive at k = 10 at the smaller size; null in all 2 at the larger size**	negative in all 2	—
Corpus/eval split	2 added (seeds 43, 44)	null in all 2	null in all 2	**negative in all 2 at the smaller size; null at seed 43, negative at seed 44 at the larger size**	positive in all 2
Retriever	1 (gte-large)	null	null	negative	—
Detector	1 (rolling z-score)	null	null	**negative at the smaller size; null at the larger size**	positive
Generator family	1 (Llama 8B and 70B)	null	null	**null at the smaller size; negative at the larger size**	positive
Label-free threshold	3 (alarm rates 0.02, 0.05, 0.10)	**negative in all 3 at the smaller size; negative at rate 0.02 and rate 0.05, null at rate 0.10 at the larger size**	null in all 3	negative in all 3	**null in all 3 at the smaller size; null at rate 0.02 and rate 0.05, positive at rate 0.10 at the larger size**
Fixed-recall target (preregistered)	2 (recall 0.7, 0.5)	null in all 2	**positive in all 2**	negative in all 2	**positive at recall 0.7, null at recall 0.5**

**Table 4:** Candidate set composition at each operating point, with the 72B LLM increment on F1 that the candidate set supports, and the same increment restricted to the 19 SKAB industrial process streams. Two families are shown and should not be read as one curve: the **fixed-recall grid** holds the thresholding policy at the oracle and varies only the recall target, which is the preregistered test of whether the increment tracks candidate recall; the **label-free rates** change the policy itself, so candidate recall and candidate precision move together there and recall is not isolated. Detector columns are stream-level macro-averages (F1 averaged per stream) over the same 40 evaluation streams. Every row except the frozen configuration was added at revision; verdicts state whether the bootstrap interval excludes zero and carry no multiplicity correction.

Family	Operating Point	Cand. Precision	Cand. Recall	Cand. F1	Cand. Events	72B LLM ΔF1 [95% CI]	72B LLM ΔF1, SKAB only [95% CI]
Fixed-recall grid (policy fixed)	Oracle, recall = 0.9 (frozen)	0.269	0.978	0.394	896	+0.0502 [+0.0284, +0.0775]	+0.0199 [+0.0129, +0.0268]
Fixed-recall grid (policy fixed)	Oracle, recall = 0.7	0.321	0.827	0.427	1785	+0.0227 [+0.0085, +0.0380]	+0.0057 [−0.0058, +0.0174] (null)
Fixed-recall grid (policy fixed)	Oracle, recall = 0.5	0.382	0.622	0.444	1590	+0.0130 [−0.0007, +0.0296] (null)	−0.0068 [−0.0166, +0.0019] (null)
Label-free alarm rate (policy varies)	Alarm rate 0.10	0.371	0.263	0.288	888	+0.0081 [+0.0025, +0.0150]	+0.0000 [+0.0000, +0.0000] (identical to detector)
Label-free alarm rate (policy varies)	Alarm rate 0.05	0.401	0.154	0.211	452	−0.0045 [−0.0116, +0.0010] (null)	−0.0014 [−0.0043, +0.0001] (null)
Label-free alarm rate (policy varies)	Alarm rate 0.02	0.380	0.069	0.114	172	−0.0036 [−0.0111, +0.0008] (null)	+0.0000 [+0.0000, +0.0000] (identical to detector)

**Table 5:** Per-candidate-alarm latency by pipeline stage, measured on one DGX Spark GB10 node with the serving configuration used throughout the study. Retrieval figures are over 60 queries and generator figures over 40 alarms. The reranking row includes the retrieval stage it depends on, since reranking cannot run without it; the two rows are therefore not additive. Index construction is a one-off cost paid before the stream is processed, not per alarm.

Stage	Mean	Median	p90
Retrieval, k = 5	14.9 ms	14.1 ms	17.3 ms
Retrieval + cross-encoder reranking (incl. retrieval)	68.3 ms	66.7 ms	81.7 ms
LLM triage, 7B	2.93 s	2.87 s	3.46 s
LLM triage, 72B	32.72 s	31.22 s	38.72 s
Retrieval index construction (one-off, 78 cases)	27.1 s	—	—

## Data Availability

This study uses two publicly available benchmarks: the Numenta Anomaly Benchmark (NAB) and the Skoltech Anomaly Benchmark (SKAB), both used in their official, unmodified form. The analysis scripts, the build verification test suite, the processed result tables, the SHA-256-frozen preregistration, the preregistration amendment and the scripts and per-stream results for every reported sensitivity axis are archived at Zenodo, DOI: 10.5281/zenodo.21487221 (https://doi.org/10.5281/zenodo.21487221), a concept DOI that always resolves to the latest version. The preregistration fixes all script hashes, model identifiers, decoding parameters, retrieval configuration, arm and increment definitions and the primary contrast decision rule prior to result computation. This archive also serves as the preregistration record for the study.

## Supplementary Materials

The following supporting information can be downloaded at https://www.mdpi.com/article/10.3390/s26165246/s1: Table S0: The frozen configuration for reference, including the PA-F1 and faithfulness increments. Tables S1–S12: Table S1: Label-free candidate threshold, alarm rate 0.02. Table S2: Label-free candidate threshold, alarm rate 0.05. Table S3: Label-free candidate threshold, alarm rate 0.10. Table S4: Neighbor count k = 1. Table S5: Neighbor count k = 10. Table S6: Alternative retriever. Table S7: Alternative detector. Table S8: Second generator family. Table S9: Corpus/eval split, seed 43. Table S10: Corpus/eval split, seed 44. Table S11: Fixed-recall grid, candidate recall = 0.7. Table S12: Fixed-recall grid, candidate recall = 0.5. Per-axis detail for every sensitivity analysis summarized in Table 3. Table S13: The per-stream distribution of each increment. Table S14: The Benjamini–Hochberg correction for the secondary increment family. Table S15: The primary contrast stratified by benchmark family. Table S16: Sign tests over the streams whose increment is non-zero. Table S17: The minimum detectable effect for the primary contrast in each of the thirteen configurations. Table S18: The per-alarm latency measurements. Table S19: The strand-level coding of the prior systems reviewed in Section 2.1. All supplementary tables except Table S19 were generated directly from the released result files; Table S19 records a manual coding of the cited literature, with the search terms and hit counts reported alongside it.

## Appendix A. Prompt and Description Templates

Prompt design is not varied in this study, so the exact templates are reproduced here verbatim. They are identical across configurations and generator sizes; the only difference between arms is whether the retrieved-cases block is present, and, for the reranking arm, how the retrieved cases are ordered. Decoding was greedy (temperature 0, seed 42, 256-token cap) for every call. Line breaks inside the boxes below are for display only where the underlying string is a single line.

The system prompt, held fixed for all calls:

You are an industrial IoT monitoring assistant. A statistical
  detector has flagged a candidate anomaly. Decide whether to
  CONFIRM it as a real anomaly or SUPPRESS it as a likely false
  alarm, and explain briefly. Respond as JSON:
  {“decision”: “confirm”|“suppress”, “explanation”: “...”}.

The user message. The detector-only arm never reaches the generator; the llm arm omits the retrieved-cases block entirely; the llm_retrieval and llm_rerank arms include it with *k* cases:

Candidate anomaly:
  {window_description}

Similar past anomaly cases (retrieved):     <- omitted in the llm arm
  - {case_1_description}
  - ...
  - {case_k_description}

Return the JSON decision.

Both {window_description} and {case_i_description} are produced by one deterministic renderer, so query and corpus text are drawn from the same vocabulary. The percentile clause is present for candidate windows and absent for corpus cases:

Anomaly in {benchmark} stream {series_id}. Duration {n} steps.
  Most deviating channels: {ch1} (peak {z1} sigma), {ch2} (peak {z2} sigma),
  {ch3} (peak {z3} sigma). Peak deviation {z1} sigma on {ch1}.
  Detector anomaly score at the {p}th percentile.

Channels are ranked by peak absolute deviation within the window, standardized against that channel’s full-series mean and standard deviation, and the top three are listed.

Faithfulness scoring (SQ4) reuses these same strings rather than a separate rendering. The premise is the window description, with the retrieved-case texts appended for the retrieval arms. The generator’s explanation is split into sentence-level claims, and each claim is scored against the premise by the local NLI model:

premise = {window_description}
        [ + “ Retrieved past cases: ”
         + {case_1_description} ... {case_k_description} ]
  claims  = sentence split of the explanation field,
        keeping fragments longer than 8 characters
  score  = mean entailment probability over (premise, claim)

This construction is deliberately strict; a claim that is plausible but not stated in the premise scores low, which is why absolute entailment values sit near the floor and are interpreted only comparatively across arms.
