# Supplementary material for: Component-Level Contributions of Retrieval-Augmented LLM Post-Processing in Streaming Anomaly Detection: A Matched-Operating-Point Case Audit
Source: Sensors (Basel). 2026 Aug 19;26(16):5246. doi: 10.3390/s26165246 (PMC13517787; doi:10.3390/s26165246)
Supplement: Supplementary file 1 [file sensors-26-05246-s001.zip › sensors-4484589-supplementary.pdf]

Supplementary Material

Supplementary Material

Per-axis detail for the sensitivity analyses summarized in Table 3 of the main text. Every table here except S19 is generated directly from the result files released with the study; no value is transcribed by hand. All intervals are stream-level bootstrap confidence intervals (B = 10,000, seed 42, 2.5/97.5 percentiles), computed exactly as in the main analysis. Each axis changes one factor against the frozen configuration and leaves every other preregistered setting untouched.

S1-S10 are exploratory axes, declared in the preregistration amendment before each run was executed; they do not alter the preregistered primary contrast. S11-S12 are different: those operating points were preregistered in the original SHA-256 freeze and left unexecuted until revision, and they break the preregistered null on precision at both targets (Section 4.7).

S0. Frozen configuration (reference)

The preregistered configuration against which every axis below is compared.

Generator	Metric	Increment	n	Mean Δ	95% CI	Verdict
72B	F1	LLM	40	0.0502	[0.0284, 0.0775]	positive
72B	Precision	LLM	40	0.0420	[0.0250, 0.0640]	positive
72B	Recall	LLM	40	−0.0333	[−0.0731, −0.0082]	negative
72B	PA-F1	LLM	40	0.0557	[0.0323, 0.0844]	positive
72B	Faithfulness	LLM	40	0.0234	[0.0137, 0.0343]	positive
72B	F1	Retrieval	40	0.0095	[−0.0062, 0.0231]	null
72B	Precision	Retrieval	40	0.0079	[−0.0034, 0.0179]	null
72B	Recall	Retrieval	40	−0.0288	[−0.0602, −0.0049]	negative
72B	PA-F1	Retrieval	40	0.0129	[−0.0040, 0.0292]	null
72B	Faithfulness	Retrieval	40	−0.0013	[−0.0121, 0.0098]	null
72B	F1	Rerank	40	−0.0067	[−0.0134, −9.0e−04]	negative
72B	Precision	Rerank	40	−0.0052	[−0.0103, −9.0e−04]	negative
72B	Recall	Rerank	40	−4.0e−04	[−0.0028, 0.0019]	null
72B	PA-F1	Rerank	40	−0.0066	[−0.0137, −7.0e−04]	negative
72B	Faithfulness	Rerank	40	−0.0032	[−0.0108, 0.0034]	null
7B	F1	LLM	40	0.0108	[0.0048, 0.0187]	positive
7B	Precision	LLM	40	0.0079	[0.0039, 0.0134]	positive
7B	Recall	LLM	40	−0.0083	[−0.0150, −0.0028]	negative
7B	PA-F1	LLM	40	0.0129	[0.0070, 0.0208]	positive
7B	Faithfulness	LLM	40	0.0419	[0.0192, 0.0727]	positive
7B	F1	Retrieval	40	−0.0122	[−0.0585, 0.0252]	null
7B	Precision	Retrieval	40	9.0e−04	[−0.0315, 0.0280]	null
7B	Recall	Retrieval	40	−0.2063	[−0.3185, −0.1043]	negative
7B	PA-F1	Retrieval	40	0.0110	[−0.0215, 0.0427]	null
7B	Faithfulness	Retrieval	40	−0.0139	[−0.0473, 0.0109]	null
7B	F1	Rerank	40	0.0143	[−0.0420, 0.0759]	null
7B	Precision	Rerank	40	0.0093	[−0.0290, 0.0529]	null
7B	Recall	Rerank	40	0.0484	[−0.0742, 0.1686]	null
7B	PA-F1	Rerank	40	−0.0031	[−0.0528, 0.0494]	null
7B	Faithfulness	Rerank	40	0.0077	[0.0012, 0.0138]	positive

S1. Label-free candidate threshold, alarm rate 0.02

The candidate threshold is set at a fixed 2% alarm rate, using no labels, in place of the oracle threshold fixed at 0.9 recall.

Generator	Metric	Increment	n	Mean Δ	95% CI	Verdict
72B	F1	LLM	40	−0.0036	[−0.0111, 8.1e−04]	null
72B	Precision	LLM	40	0.0046	[−0.0030, 0.0163]	null
72B	Recall	LLM	40	−0.0029	[−0.0080, 0.0000]	null
72B	Faithfulness	LLM	40	0.0112	[0.0032, 0.0217]	positive
72B	F1	Retrieval	40	−0.0152	[−0.0364, −3.5e−04]	negative
72B	Precision	Retrieval	40	−0.0054	[−0.0569, 0.0327]	null
72B	Recall	Retrieval	40	−0.0095	[−0.0224, −4.2e−04]	negative
72B	Faithfulness	Retrieval	40	0.0079	[−0.0022, 0.0183]	null
72B	F1	Rerank	40	0.0000	[0.0000, 0.0000]	identical
72B	Precision	Rerank	40	0.0000	[0.0000, 0.0000]	identical
72B	Recall	Rerank	40	0.0000	[0.0000, 0.0000]	identical
72B	Faithfulness	Rerank	40	3.0e−04	[−0.0078, 0.0085]	null
7B	F1	LLM	40	0.0000	[0.0000, 0.0000]	identical
7B	Precision	LLM	40	0.0000	[0.0000, 0.0000]	identical
7B	Recall	LLM	40	0.0000	[0.0000, 0.0000]	identical
7B	Faithfulness	LLM	40	0.0310	[0.0130, 0.0525]	positive
7B	F1	Retrieval	40	−0.0394	[−0.0703, −0.0131]	negative
7B	Precision	Retrieval	40	−0.0189	[−0.0922, 0.0429]	null
7B	Recall	Retrieval	40	−0.0254	[−0.0448, −0.0089]	negative
7B	Faithfulness	Retrieval	40	−0.0096	[−0.0333, 0.0105]	null
7B	F1	Rerank	40	0.0048	[−0.0177, 0.0287]	null
7B	Precision	Rerank	40	−0.0393	[−0.1001, 0.0076]	null
7B	Recall	Rerank	40	0.0032	[−0.0108, 0.0180]	null
7B	Faithfulness	Rerank	40	0.0014	[−0.0061, 0.0093]	null

S2. Label-free candidate threshold, alarm rate 0.05

As above at a 5% alarm rate. This is the rate declared in the original amendment; 0.02 and 0.10 were added afterwards so the finding would not rest on one threshold.

Generator	Metric	Increment	n	Mean Δ	95% CI	Verdict
72B	F1	LLM	40	−0.0045	[−0.0116, 0.0010]	null
72B	Precision	LLM	40	0.0256	[0.0046, 0.0520]	positive
72B	Recall	LLM	40	−0.0105	[−0.0208, −0.0026]	negative
72B	Faithfulness	LLM	40	0.0184	[0.0095, 0.0289]	positive
72B	F1	Retrieval	40	−0.0176	[−0.0401, −4.0e−04]	negative
72B	Precision	Retrieval	40	0.0127	[−0.0175, 0.0432]	null
72B	Recall	Retrieval	40	−0.0164	[−0.0325, −0.0038]	negative
72B	Faithfulness	Retrieval	40	0.0018	[−0.0079, 0.0107]	null
72B	F1	Rerank	40	−2.0e−04	[−8.7e−04, 6.9e−04]	null
72B	Precision	Rerank	40	−0.0049	[−0.0136, 7.2e−04]	null
72B	Recall	Rerank	40	3.0e−04	[0.0000, 7.6e−04]	null
72B	Faithfulness	Rerank	40	−0.0034	[−0.0112, 0.0042]	null
7B	F1	LLM	40	−0.0019	[−0.0050, 9.5e−05]	null
7B	Precision	LLM	40	−0.0012	[−0.0033, 3.6e−04]	null
7B	Recall	LLM	40	−0.0019	[−0.0048, 0.0000]	null
7B	Faithfulness	LLM	40	0.0336	[0.0197, 0.0489]	positive
7B	F1	Retrieval	40	−0.0378	[−0.0707, −0.0112]	negative
7B	Precision	Retrieval	40	−6.0e−04	[−0.0489, 0.0435]	null
7B	Recall	Retrieval	40	−0.0361	[−0.0634, −0.0136]	negative
7B	Faithfulness	Retrieval	40	−0.0081	[−0.0247, 0.0075]	null
7B	F1	Rerank	40	1.0e−04	[−0.0312, 0.0282]	null
7B	Precision	Rerank	40	−0.0211	[−0.0623, 0.0107]	null
7B	Recall	Rerank	40	4.0e−04	[−0.0232, 0.0216]	null

Generator	Metric	Increment	n	Mean Δ	95% CI	Verdict
7B	Faithfulness	Rerank	40	0.0015	[−0.0071, 0.0109]	null

S3. Label-free candidate threshold, alarm rate 0.10

As above at a 10% alarm rate, the most permissive budget tested.

Generator	Metric	Increment	n	Mean Δ	95% CI	Verdict
72B	F1	LLM	40	0.0081	[0.0025, 0.0150]	positive
72B	Precision	LLM	40	0.0337	[0.0106, 0.0723]	positive
72B	Recall	LLM	40	−0.0085	[−0.0166, −0.0023]	negative
72B	Faithfulness	LLM	40	0.0211	[0.0123, 0.0308]	positive
72B	F1	Retrieval	40	−0.0150	[−0.0353, 0.0021]	null
72B	Precision	Retrieval	40	0.0280	[−0.0081, 0.0681]	null
72B	Recall	Retrieval	40	−0.0298	[−0.0518, −0.0118]	negative
72B	Faithfulness	Retrieval	40	0.0024	[−0.0084, 0.0136]	null
72B	F1	Rerank	40	−0.0057	[−0.0147, 0.0013]	null
72B	Precision	Rerank	40	−0.0096	[−0.0255, 0.0026]	null
72B	Recall	Rerank	40	−0.0030	[−0.0106, 0.0020]	null
72B	Faithfulness	Rerank	40	0.0070	[−0.0026, 0.0179]	null
7B	F1	LLM	40	2.0e−04	[−0.0031, 0.0025]	null
7B	Precision	LLM	40	0.0020	[−0.0011, 0.0049]	null
7B	Recall	LLM	40	−0.0021	[−0.0057, 0.0000]	null
7B	Faithfulness	LLM	40	0.0293	[0.0159, 0.0447]	positive
7B	F1	Retrieval	40	−0.0394	[−0.0744, −0.0088]	negative
7B	Precision	Retrieval	40	0.0217	[−0.0315, 0.0801]	null
7B	Recall	Retrieval	40	−0.0619	[−0.0965, −0.0324]	negative
7B	Faithfulness	Retrieval	40	−0.0052	[−0.0206, 0.0087]	null
7B	F1	Rerank	40	0.0068	[−0.0265, 0.0393]	null
7B	Precision	Rerank	40	−0.0240	[−0.0622, 0.0095]	null
7B	Recall	Rerank	40	0.0099	[−0.0274, 0.0455]	null
7B	Faithfulness	Rerank	40	0.0045	[−0.0025, 0.0118]	null

S4. Neighbor count k = 1

Retrieval returns a single neighbor instead of five.

Generator	Metric	Increment	n	Mean Δ	95% CI	Verdict
72B	F1	LLM	40	0.0502	[0.0284, 0.0775]	positive
72B	Precision	LLM	40	0.0420	[0.0247, 0.0633]	positive
72B	Recall	LLM	40	−0.0333	[−0.0719, −0.0087]	negative
72B	Faithfulness	LLM	40	0.0234	[0.0136, 0.0342]	positive
72B	F1	Retrieval	40	−0.0079	[−0.0209, 0.0015]	null
72B	Precision	Retrieval	40	−0.0071	[−0.0168, 3.1e−04]	null
72B	Recall	Retrieval	40	−0.0272	[−0.0602, −0.0035]	negative
72B	Faithfulness	Retrieval	40	0.0060	[−0.0033, 0.0156]	null
7B	F1	LLM	40	0.0108	[0.0048, 0.0187]	positive
7B	Precision	LLM	40	0.0079	[0.0039, 0.0133]	positive
7B	Recall	LLM	40	−0.0083	[−0.0152, −0.0030]	negative
7B	Faithfulness	LLM	40	0.0419	[0.0191, 0.0727]	positive
7B	F1	Retrieval	40	0.0069	[−0.0313, 0.0447]	null
7B	Precision	Retrieval	40	0.0197	[−0.0083, 0.0507]	null
7B	Recall	Retrieval	40	−0.1917	[−0.3085, −0.0916]	negative
7B	Faithfulness	Retrieval	40	−0.0282	[−0.0603, −0.0034]	negative

S5. Neighbor count k = 10

Retrieval returns ten neighbors instead of five.

Generator	Metric	Increment	n	Mean Δ	95% CI	Verdict
72B	F1	LLM	40	0.0502	[0.0284, 0.0775]	positive
72B	Precision	LLM	40	0.0420	[0.0247, 0.0633]	positive
72B	Recall	LLM	40	−0.0333	[−0.0719, −0.0087]	negative
72B	Faithfulness	LLM	40	0.0234	[0.0136, 0.0342]	positive
72B	F1	Retrieval	40	0.0068	[−0.0077, 0.0182]	null
72B	Precision	Retrieval	40	0.0070	[−0.0037, 0.0159]	null
72B	Recall	Retrieval	40	−0.0288	[−0.0614, −0.0048]	negative
72B	Faithfulness	Retrieval	40	−0.0038	[−0.0142, 0.0058]	null
7B	F1	LLM	40	0.0108	[0.0048, 0.0187]	positive
7B	Precision	LLM	40	0.0079	[0.0039, 0.0133]	positive
7B	Recall	LLM	40	−0.0083	[−0.0152, −0.0030]	negative
7B	Faithfulness	LLM	40	0.0419	[0.0191, 0.0727]	positive
7B	F1	Retrieval	40	0.0266	[−0.0038, 0.0594]	null
7B	Precision	Retrieval	40	0.0259	[0.0029, 0.0521]	positive
7B	Recall	Retrieval	40	−0.1009	[−0.1807, −0.0340]	negative
7B	Faithfulness	Retrieval	40	0.0081	[−0.0252, 0.0346]	null

S6. Alternative retriever

The embedding model is gte-large in place of bge-large-en-v1.5.

Generator	Metric	Increment	n	Mean Δ	95% CI	Verdict
72B	F1	LLM	40	0.0502	[0.0284, 0.0775]	positive
72B	Precision	LLM	40	0.0420	[0.0247, 0.0633]	positive
72B	Recall	LLM	40	−0.0333	[−0.0719, −0.0087]	negative
72B	Faithfulness	LLM	40	0.0234	[0.0136, 0.0342]	positive
72B	F1	Retrieval	40	0.0189	[−0.0030, 0.0451]	null
72B	Precision	Retrieval	40	0.0159	[−6.3e−04, 0.0373]	null
72B	Recall	Retrieval	40	−0.0287	[−0.0612, −0.0047]	negative
72B	Faithfulness	Retrieval	40	−0.0010	[−0.0119, 0.0099]	null
7B	F1	LLM	40	0.0108	[0.0048, 0.0187]	positive
7B	Precision	LLM	40	0.0079	[0.0039, 0.0133]	positive
7B	Recall	LLM	40	−0.0083	[−0.0152, −0.0030]	negative
7B	Faithfulness	LLM	40	0.0419	[0.0191, 0.0727]	positive
7B	F1	Retrieval	40	−0.0043	[−0.0545, 0.0403]	null
7B	Precision	Retrieval	40	0.0073	[−0.0293, 0.0414]	null
7B	Recall	Retrieval	40	−0.1770	[−0.2891, −0.0779]	negative
7B	Faithfulness	Retrieval	40	−0.0110	[−0.0432, 0.0140]	null

S7. Alternative detector

Candidate events come from a causal rolling z-score detector instead of IsolationForest.

Generator	Metric	Increment	n	Mean Δ	95% CI	Verdict
72B	F1	LLM	40	0.0122	[0.0017, 0.0262]	positive
72B	Precision	LLM	40	0.0083	[9.6e−04, 0.0178]	positive
72B	Recall	LLM	40	−0.0062	[−0.0175, 0.0000]	null
72B	Faithfulness	LLM	40	0.0187	[0.0040, 0.0442]	positive
72B	F1	Retrieval	40	−0.0029	[−0.0162, 0.0052]	null
72B	Precision	Retrieval	40	−7.0e−04	[−0.0089, 0.0052]	null
72B	Recall	Retrieval	40	−0.0401	[−0.1052, 0.0000]	null
72B	Faithfulness	Retrieval	40	−0.0080	[−0.0271, 0.0034]	null
72B	F1	Rerank	40	1.0e−04	[0.0000, 2.8e−04]	null
72B	Precision	Rerank	40	2.0e−04	[0.0000, 4.4e−04]	null
72B	Recall	Rerank	40	−0.0048	[−0.0145, 0.0000]	null
72B	Faithfulness	Rerank	40	−0.0016	[−0.0042, 6.0e−04]	null
7B	F1	LLM	40	4.0e−04	[3.0e−06, 9.8e−04]	positive
7B	Precision	LLM	40	2.0e−04	[3.0e−06, 6.3e−04]	positive
7B	Recall	LLM	40	0.0000	[0.0000, 0.0000]	identical
7B	Faithfulness	LLM	40	0.0672	[0.0253, 0.1167]	positive
7B	F1	Retrieval	40	−0.0162	[−0.0364, 0.0018]	null

Generator	Metric	Increment	n	Mean Δ	95% CI	Verdict
7B	Precision	Retrieval	40	−0.0079	[−0.0193, 0.0028]	null
7B	Recall	Retrieval	40	−0.1435	[−0.2504, −0.0534]	negative
7B	Faithfulness	Retrieval	40	−0.0490	[−0.0995, −0.0055]	negative
7B	F1	Rerank	40	0.0058	[−0.0197, 0.0342]	null
7B	Precision	Rerank	40	0.0037	[−0.0108, 0.0205]	null
7B	Recall	Rerank	40	0.0123	[−0.1083, 0.1301]	null
7B	Faithfulness	Rerank	40	0.0088	[−0.0055, 0.0254]	null

S8. Second generator family

Qwen2.5-7B/72B are replaced by Llama-3.1-8B and Llama-3.3-70B, served identically (llama.cpp, Q8_0, temperature 0, seed 42).

Generator	Metric	Increment	n	Mean Δ	95% CI	Verdict
Llama 70B	F1	LLM	40	0.0548	[0.0322, 0.0825]	positive
Llama 70B	Precision	LLM	40	0.0483	[0.0297, 0.0713]	positive
Llama 70B	Recall	LLM	40	−0.0376	[−0.0771, −0.0114]	negative
Llama 70B	Faithfulness	LLM	40	0.0149	[0.0066, 0.0262]	positive
Llama 70B	F1	Retrieval	40	0.0092	[−0.0137, 0.0379]	null
Llama 70B	Precision	Retrieval	40	0.0150	[−0.0086, 0.0486]	null
Llama 70B	Recall	Retrieval	40	−0.0556	[−0.1061, −0.0128]	negative
Llama 70B	Faithfulness	Retrieval	40	0.0226	[0.0076, 0.0404]	positive
Llama 70B	F1	Rerank	40	−0.0096	[−0.0336, 0.0094]	null
Llama 70B	Precision	Rerank	40	−0.0178	[−0.0479, 0.0026]	null
Llama 70B	Recall	Rerank	40	0.0471	[0.0090, 0.0944]	positive
Llama 70B	Faithfulness	Rerank	40	0.0049	[−0.0128, 0.0209]	null
Llama 8B	F1	LLM	40	0.0436	[0.0194, 0.0739]	positive
Llama 8B	Precision	LLM	40	0.0385	[0.0183, 0.0632]	positive
Llama 8B	Recall	LLM	40	−0.0455	[−0.0858, −0.0169]	negative
Llama 8B	Faithfulness	LLM	40	0.0193	[0.0106, 0.0294]	positive
Llama 8B	F1	Retrieval	40	−0.0062	[−0.0285, 0.0155]	null
Llama 8B	Precision	Retrieval	40	−0.0102	[−0.0290, 0.0078]	null
Llama 8B	Recall	Retrieval	40	0.0180	[−0.0078, 0.0488]	null
Llama 8B	Faithfulness	Retrieval	40	0.0037	[−0.0090, 0.0165]	null
Llama 8B	F1	Rerank	40	−0.0056	[−0.0202, 0.0079]	null
Llama 8B	Precision	Rerank	40	−0.0048	[−0.0158, 0.0052]	null
Llama 8B	Recall	Rerank	40	0.0093	[−0.0057, 0.0274]	null
Llama 8B	Faithfulness	Rerank	40	0.0024	[−0.0054, 0.0095]	null

S9. Corpus/eval split, seed 43

The corpus/eval series split is redrawn with seed 43.

Generator	Metric	Increment	n	Mean Δ	95% CI	Verdict
72B	F1	LLM	40	0.0349	[0.0174, 0.0584]	positive
72B	Precision	LLM	40	0.0290	[0.0152, 0.0487]	positive
72B	Recall	LLM	40	−0.0199	[−0.0347, −0.0080]	negative
72B	Faithfulness	LLM	40	0.0179	[0.0090, 0.0283]	positive
72B	F1	Retrieval	40	0.0041	[−0.0022, 0.0121]	null
72B	Precision	Retrieval	40	0.0027	[−0.0015, 0.0084]	null
72B	Recall	Retrieval	40	−0.0080	[−0.0229, 0.0000]	null
72B	Faithfulness	Retrieval	40	0.0010	[−0.0078, 0.0094]	null
72B	F1	Rerank	40	−2.0e−04	[−0.0033, 0.0035]	null
72B	Precision	Rerank	40	9.0e−04	[−0.0023, 0.0045]	null
72B	Recall	Rerank	40	−0.0113	[−0.0349, 9.1e−04]	null
72B	Faithfulness	Rerank	40	−3.0e−04	[−0.0060, 0.0051]	null
7B	F1	LLM	40	0.0059	[0.0015, 0.0110]	positive
7B	Precision	LLM	40	0.0049	[0.0019, 0.0084]	positive
7B	Recall	LLM	40	−0.0142	[−0.0250, −0.0051]	negative
7B	Faithfulness	LLM	40	0.0480	[0.0183, 0.0857]	positive
7B	F1	Retrieval	40	−0.0155	[−0.0768, 0.0475]	null

Generator	Metric	Increment	n	Mean Δ	95% CI	Verdict
7B	Precision	Retrieval	40	0.0075	[−0.0429, 0.0661]	null
7B	Recall	Retrieval	40	−0.1918	[−0.3075, −0.0913]	negative
7B	Faithfulness	Retrieval	40	−0.0208	[−0.0617, 0.0099]	null
7B	F1	Rerank	40	0.0103	[−0.0461, 0.0706]	null
7B	Precision	Rerank	40	−0.0026	[−0.0537, 0.0459]	null
7B	Recall	Rerank	40	0.0436	[−0.0709, 0.1559]	null
7B	Faithfulness	Rerank	40	3.0e−04	[−0.0064, 0.0070]	null

S10. Corpus/eval split, seed 44

The corpus/eval series split is redrawn with seed 44.

Generator	Metric	Increment	n	Mean Δ	95% CI	Verdict
72B	F1	LLM	40	0.0604	[0.0350, 0.0896]	positive
72B	Precision	LLM	40	0.0497	[0.0290, 0.0742]	positive
72B	Recall	LLM	40	−0.0439	[−0.0843, −0.0158]	negative
72B	Faithfulness	LLM	40	0.0099	[0.0043, 0.0170]	positive
72B	F1	Retrieval	40	0.0044	[−0.0084, 0.0151]	null
72B	Precision	Retrieval	40	0.0055	[−0.0027, 0.0130]	null
72B	Recall	Retrieval	40	−0.0490	[−0.1127, −0.0064]	negative
72B	Faithfulness	Retrieval	40	0.0048	[−0.0039, 0.0129]	null
72B	F1	Rerank	40	−0.0065	[−0.0209, 0.0062]	null
72B	Precision	Rerank	40	−0.0021	[−0.0127, 0.0074]	null
72B	Recall	Rerank	40	−0.0157	[−0.0404, 0.0013]	null
72B	Faithfulness	Rerank	40	−8.0e−04	[−0.0057, 0.0037]	null
7B	F1	LLM	40	0.0134	[0.0066, 0.0219]	positive
7B	Precision	LLM	40	0.0094	[0.0047, 0.0152]	positive
7B	Recall	LLM	40	−0.0153	[−0.0250, −0.0071]	negative
7B	Faithfulness	LLM	40	0.0383	[0.0161, 0.0679]	positive
7B	F1	Retrieval	40	−0.0259	[−0.0785, 0.0229]	null
7B	Precision	Retrieval	40	0.0142	[−0.0334, 0.0676]	null
7B	Recall	Retrieval	40	−0.3068	[−0.4323, −0.1882]	negative
7B	Faithfulness	Retrieval	40	−0.0086	[−0.0402, 0.0154]	null
7B	F1	Rerank	40	0.0165	[−0.0156, 0.0552]	null
7B	Precision	Rerank	40	−0.0055	[−0.0524, 0.0398]	null
7B	Recall	Rerank	40	0.0340	[−0.0192, 0.0964]	null
7B	Faithfulness	Rerank	40	−0.0026	[−0.0085, 0.0032]	null

S11. Fixed-recall grid, candidate recall = 0.7

The thresholding policy stays at the oracle and only the recall target moves, so candidate recall is isolated from the policy change that the label-free rates also introduce. Preregistered in the original freeze (Section 3.4) and executed at revision.

Generator	Metric	Increment	n	Mean Δ	95% CI	Verdict
72B	F1	LLM	40	0.0227	[0.0085, 0.0380]	positive
72B	Precision	LLM	40	0.0296	[0.0172, 0.0458]	positive
72B	Recall	LLM	40	−0.0765	[−0.1221, −0.0413]	negative
72B	Faithfulness	LLM	40	0.0297	[0.0179, 0.0433]	positive
72B	F1	Retrieval	40	0.0063	[−0.0119, 0.0246]	null
72B	Precision	Retrieval	40	0.0148	[0.0013, 0.0301]	positive
72B	Recall	Retrieval	40	−0.0448	[−0.0789, −0.0173]	negative
72B	Faithfulness	Retrieval	40	−0.0049	[−0.0173, 0.0066]	null
72B	F1	Rerank	40	0.0038	[−0.0120, 0.0219]	null
72B	Precision	Rerank	40	−0.0022	[−0.0115, 0.0058]	null
72B	Recall	Rerank	40	0.0147	[−0.0171, 0.0493]	null
72B	Faithfulness	Rerank	40	0.0052	[−0.0021, 0.0124]	null
7B	F1	LLM	40	0.0079	[0.0037, 0.0123]	positive
7B	Precision	LLM	40	0.0065	[0.0036, 0.0098]	positive
7B	Recall	LLM	40	−0.0169	[−0.0260, −0.0092]	negative
7B	Faithfulness	LLM	40	0.0294	[0.0175, 0.0425]	positive

Generator	Metric	Increment	n	Mean Δ	95% CI	Verdict
7B	F1	Retrieval	40	0.0178	[−0.0150, 0.0486]	null
7B	Precision	Retrieval	40	0.0388	[0.0090, 0.0710]	positive
7B	Recall	Retrieval	40	−0.1312	[−0.2043, −0.0668]	negative
7B	Faithfulness	Retrieval	40	−1.0e−04	[−0.0153, 0.0148]	null
7B	F1	Rerank	40	0.0000	[−0.0352, 0.0327]	null
7B	Precision	Rerank	40	0.0146	[−0.0348, 0.0791]	null
7B	Recall	Rerank	40	0.0174	[−0.0432, 0.0784]	null
7B	Faithfulness	Rerank	40	0.0097	[0.0012, 0.0183]	positive

S12. Fixed-recall grid, candidate recall = 0.5

As above at a 0.5 recall target, the lowest point of the preregistered grid.

Generator	Metric	Increment	n	Mean Δ	95% CI	Verdict
72B	F1	LLM	40	0.0130	[−6.6e−04, 0.0296]	null
72B	Precision	LLM	40	0.0389	[0.0108, 0.0848]	positive
72B	Recall	LLM	40	−0.0519	[−0.0779, −0.0307]	negative
72B	Faithfulness	LLM	40	0.0285	[0.0189, 0.0395]	positive
72B	F1	Retrieval	40	−0.0060	[−0.0204, 0.0067]	null
72B	Precision	Retrieval	40	0.0310	[0.0048, 0.0660]	positive
72B	Recall	Retrieval	40	−0.0367	[−0.0615, −0.0148]	negative
72B	Faithfulness	Retrieval	40	−3.0e−04	[−0.0096, 0.0081]	null
72B	F1	Rerank	40	2.0e−04	[−0.0081, 0.0075]	null
72B	Precision	Rerank	40	−8.0e−04	[−0.0087, 0.0072]	null
72B	Recall	Rerank	40	0.0042	[−0.0070, 0.0143]	null
72B	Faithfulness	Rerank	40	7.0e−04	[−0.0050, 0.0058]	null
7B	F1	LLM	40	0.0019	[−9.8e−04, 0.0048]	null
7B	Precision	LLM	40	0.0031	[5.2e−04, 0.0062]	positive
7B	Recall	LLM	40	−0.0101	[−0.0169, −0.0046]	negative
7B	Faithfulness	LLM	40	0.0304	[0.0187, 0.0436]	positive
7B	F1	Retrieval	40	−0.0118	[−0.0414, 0.0163]	null
7B	Precision	Retrieval	40	0.0639	[0.0109, 0.1263]	positive
7B	Recall	Retrieval	40	−0.1091	[−0.1585, −0.0650]	negative
7B	Faithfulness	Retrieval	40	−0.0046	[−0.0186, 0.0082]	null
7B	F1	Rerank	40	−0.0087	[−0.0398, 0.0215]	null
7B	Precision	Rerank	40	−0.0409	[−0.0831, −0.0049]	negative
7B	Recall	Rerank	40	0.0060	[−0.0475, 0.0549]	null
7B	Faithfulness	Rerank	40	0.0089	[0.0016, 0.0163]	positive

S13. Per-stream distribution of each increment

Reviewer 1 asked whether the mean increments hide heterogeneity across streams. They do. The columns below describe the distribution of the per-stream increment rather than its mean: `median` is the typical stream, `frac zero` the share of streams the increment leaves exactly unchanged, and `mean after dropping the 5 drivers` the mean recomputed with the five streams that drive the effect removed — the five largest when the mean is positive, the five smallest when it is negative, so the trim always cuts the tail responsible for the effect rather than the tail opposing it.

Generator	Metric	Increment	n	Mean	Median	Frac. zero	Mean after dropping the 5 drivers
72B	F1	LLM	40	0.0502	0.0247	0.175	0.0248
7B	F1	LLM	40	0.0108	0.0036	0.275	0.0043
72B	F1	Rerank	40	−0.0067	0.0000	0.525	−5.0e−04
7B	F1	Rerank	40	0.0143	0.0000	0.300	−0.0352
72B	F1	Retrieval	40	0.0095	0.0000	0.375	−0.0012
7B	F1	Retrieval	40	−0.0122	1.0e−04	0.275	0.0298
72B	Recall	LLM	40	−0.0333	0.0000	0.675	−0.0051
7B	Recall	LLM	40	−0.0083	0.0000	0.750	−0.0022
72B	Recall	Rerank	40	−4.0e−04	0.0000	0.925	8.0e−04
7B	Recall	Rerank	40	0.0484	0.0000	0.700	−0.0485

Generator	Metric	Increment	n	Mean	Median	Frac. zero	Mean after dropping the 5 drivers
72B	Recall	Retrieval	40	−0.0288	0.0000	0.825	−0.0010
7B	Recall	Retrieval	40	−0.2063	0.0000	0.675	−0.0996

S14. Multiplicity correction for the secondary increment family

Benjamini–Hochberg correction across the secondary increment family, with the minimum detectable effect at 80% power for reference.

Generator	Metric	Increment	Mean Δ	Bootstrap p	BH-adjusted p	Significant after BH	MDE (80%)
72B	F1	LLM	0.0502	1.0e−04	2.4e−04	yes	0.0352
72B	F1	Retrieval	0.0095	0.2090		no	0.0209
72B	F1	Rerank	−0.0067	0.0192	0.0357	yes	0.0089
72B	PA-F1	LLM	0.0557	1.0e−04	2.4e−04	yes	0.0376
72B	PA-F1	Retrieval	0.0129	0.1236	0.1890	no	0.0233
72B	PA-F1	Rerank	−0.0066	0.0232	0.0377	yes	0.0093
72B	Precision	LLM	0.0420	1.0e−04	2.4e−04	yes	0.0282
72B	Precision	Retrieval	0.0079	0.1606		no	0.0153
72B	Precision	Rerank	−0.0052	0.0144	0.0288	yes	0.0067
72B	Recall	LLM	−0.0333	1.0e−04	2.4e−04	yes	0.0480
72B	Recall	Retrieval	−0.0288	6.0e−04	0.0013	yes	0.0401
72B	Recall	Rerank	−4.0e−04	0.7436	0.8056	no	0.0032
72B	Faithfulness	LLM	0.0234	1.0e−04	2.4e−04	yes	0.0151
72B	Faithfulness	Retrieval	−0.0013	0.8206	0.8534	no	0.0156
72B	Faithfulness	Rerank	−0.0032	0.3760	0.5145	no	0.0102
7B	F1	LLM	0.0108	1.0e−04	2.4e−04	yes	0.0100
7B	F1	Retrieval	−0.0122	0.5950		no	0.0602
7B	F1	Rerank	0.0143	0.6458	0.7632	no	0.0842
7B	PA-F1	LLM	0.0129	1.0e−04	2.4e−04	yes	0.0100
7B	PA-F1	Retrieval	0.0110	0.5064	0.6270	no	0.0464
7B	PA-F1	Rerank	−0.0031	0.9126	0.9126	no	0.0731
7B	Precision	LLM	0.0079	1.0e−04	2.4e−04	yes	0.0069
7B	Precision	Retrieval	9.0e−04	0.9102		no	0.0427
7B	Precision	Rerank	0.0093	0.6776	0.7660	no	0.0585
7B	Recall	LLM	−0.0083	1.0e−04	2.4e−04	yes	0.0088
7B	Recall	Retrieval	−0.2063	1.0e−04	2.4e−04	yes	0.1534
7B	Recall	Rerank	0.0484	0.4280	0.5564	no	0.1731
7B	Faithfulness	LLM	0.0419	1.0e−04	2.4e−04	yes	0.0390
7B	Faithfulness	Retrieval	−0.0139	0.3468	0.5009	no	0.0418
7B	Faithfulness	Rerank	0.0077	0.0212	0.0367	yes	0.0091

S15. Primary contrast stratified by benchmark family

Only 19 of the 40 evaluation streams are SKAB industrial-process series. Because the article is about industrial sensing, the increments are recomputed on each family separately, with the same bootstrap construction. The preregistered primary contrast (F1 and precision) is null on both subsets as well as pooled; the recall degradation is carried by the NAB series and is absent on the industrial subset.

Subset	Generator	Metric	Increment	n	Mean Δ	95% CI	CI excludes 0
SKAB	72B	F1	Retrieval	19	0.0024	[−0.0022, 0.0076]	no
NAB	72B	F1	Retrieval	21	0.0160	[−0.0135, 0.0410]	no
all	72B	F1	Retrieval	40	0.0095	[−0.0062, 0.0231]	no
SKAB	72B	Precision	Retrieval	19	0.0026	[−0.0025, 0.0084]	no
NAB	72B	Precision	Retrieval	21	0.0127	[−0.0090, 0.0306]	no

Subset	Generator	Metric	Increment	n	Mean Δ	95% CI	CI excludes 0
all	72B	Precision	Retrieval	40	0.0079	[−0.0034, 0.0179]	no
SKAB	72B	Recall	Retrieval	19	−4.0e−04	[−0.0011, 0.0000]	no
NAB	72B	Recall	Retrieval	21	−0.0546	[−0.1124, −0.0100]	yes
all	72B	Recall	Retrieval	40	−0.0288	[−0.0602, −0.0049]	yes
SKAB	7B	F1	Retrieval	19	−0.0280	[−0.0942, 0.0076]	no
NAB	7B	F1	Retrieval	21	0.0021	[−0.0560, 0.0575]	no
all	7B	F1	Retrieval	40	−0.0122	[−0.0585, 0.0252]	no
SKAB	7B	Precision	Retrieval	19	−0.0186	[−0.0676, 0.0087]	no
NAB	7B	Precision	Retrieval	21	0.0186	[−0.0206, 0.0556]	no
all	7B	Precision	Retrieval	40	9.0e−04	[−0.0315, 0.0280]	no
SKAB	7B	Recall	Retrieval	19	−0.0483	[−0.1449, 0.0000]	no
NAB	7B	Recall	Retrieval	21	−0.3493	[−0.5179, −0.1899]	yes
all	7B	Recall	Retrieval	40	−0.2063	[−0.3185, −0.1043]	yes

S16. Sign test over streams whose increment is non-zero

The per-stream increments are zero-inflated, so the interval on the mean and the consistency of direction answer different questions. `n non-zero` is the number of streams an interval actually rests on; the p-value is an exact two-sided sign test over those streams. Reported because it cuts both ways: the retrieval increment on F1 is directionally positive more often than not, while the withdrawn reranking penalty fails this test as well.

Generator	Metric	Increment	n streams	n non-zero	Positive	Negative	Sign-test p
72B	F1	LLM	40	33	30	3	1e-06
72B	F1	Rerank	40	19	6	13	0.167068
72B	F1	Retrieval	40	25	18	7	0.043285
72B	Precision	LLM	40	33	32	1	0.0
72B	Precision	Rerank	40	19	6	13	0.167068
72B	Precision	Retrieval	40	25	18	7	0.043285
72B	Recall	LLM	40	13	0	13	0.000244
72B	Recall	Rerank	40	3	1	2	1.0
72B	Recall	Retrieval	40	7	0	7	0.015625
7B	F1	LLM	40	29	25	4	0.000104
7B	F1	Rerank	40	28	15	13	0.850554
7B	F1	Retrieval	40	29	20	9	0.061428
7B	Precision	LLM	40	28	25	3	2.7e-05
7B	Precision	Rerank	40	28	15	13	0.850554
7B	Precision	Retrieval	40	29	20	9	0.061428
7B	Recall	LLM	40	10	0	10	0.001953
7B	Recall	Rerank	40	12	8	4	0.387695
7B	Recall	Retrieval	40	13	0	13	0.000244

S17. Minimum detectable effect and null grade for the primary contrast, by configuration

A null is only as strong as the effect it could have detected. MDE is the smallest true effect this design would detect at 80% power, computed as 2.8016 × SE from the bootstrap interval. The grade compares the largest effect the interval still admits, max(|CI lower|, |CI upper|), against the LLM increment on the same metric, generator and candidate set (column `LLM reference`) — what the generator stage itself delivers, and thus the size a retrieval stage must approach to matter. `yardstick_source` in the deposited `tbl_mde_by_configuration.csv` records which value that is:

`frozen`, the oracle recall-0.9 increment, where only retrieval varies and the generator arms are reused unchanged; `own`, the configuration's own increment, where the candidate set is regenerated or the generator family changes; `own(no-frozen-tier)` for the Llama family, which has no frozen counterpart. A null is *bounded* when the interval excludes an effect that large and *undetected* when it does not. Of 42 nulls, 15 are bounded and 27 undetected. The frozen 72B configuration admits at most 0.023 F1 against an LLM increment of 0.050; the frozen 7B configuration admits 0.059 against an LLM increment of 0.011, so there the null is undetected rather than bounded.

Configuration	Generator	Metric	Mean Δ	95% CI	MDE (80%)	Largest effect admitted	LLM reference	Verdict
frozen	7B	F1	−0.0122	[−0.0585, 0.0252]	0.0598	0.0585	0.0108	null (undetected)
frozen	7B	Precision	9.0e−04	[−0.0315, 0.0280]	0.0425	0.0315	0.0079	null (undetected)
frozen	72B	F1	0.0095	[−0.0062, 0.0231]	0.0209	0.0231	0.0502	null (bounded)
frozen	72B	Precision	0.0079	[−0.0034, 0.0179]	0.0152	0.0179	0.0420	null (bounded)
c1rate	72B	F1	−0.0176	[−0.0401, −4.0e−04]	0.0284	0.0401	0.0045	negative
c1rate	72B	Precision	0.0127	[−0.0175, 0.0432]	0.0434	0.0432	0.0256	null (undetected)
c1rate	7B	F1	−0.0378	[−0.0707, −0.0112]	0.0425	0.0707	0.0019	negative
c1rate	7B	Precision	−6.0e−04	[−0.0489, 0.0435]	0.0660	0.0489	0.0012	null (undetected)
c2k1	72B	F1	−0.0079	[−0.0209, 0.0015]	0.0161	0.0209	0.0502	null (bounded)
c2k1	72B	Precision	−0.0071	[−0.0168, 3.1e−04]	0.0122	0.0168	0.0420	null (bounded)
c2k1	7B	F1	0.0069	[−0.0313, 0.0447]	0.0543	0.0447	0.0108	null (undetected)
c2k1	7B	Precision	0.0197	[−0.0083, 0.0507]	0.0421	0.0507	0.0079	null (undetected)
c2k10	72B	F1	0.0068	[−0.0077, 0.0182]	0.0185	0.0182	0.0502	null (bounded)
c2k10	72B	Precision	0.0070	[−0.0037, 0.0159]	0.0140	0.0159	0.0420	null (bounded)
c2k10	7B	F1	0.0266	[−0.0038, 0.0594]	0.0452	0.0594	0.0108	null (undetected)
c2k10	7B	Precision	0.0259	[0.0029, 0.0521]	0.0352	0.0521	0.0079	positive
c3gte	72B	F1	0.0189	[−0.0030, 0.0451]	0.0344	0.0451	0.0502	null (bounded)
c3gte	72B	Precision	0.0159	[−6.3e−04, 0.0373]	0.0271	0.0373	0.0420	null (bounded)
c3gte	7B	F1	−0.0043	[−0.0545, 0.0403]	0.0678	0.0545	0.0108	null (undetected)
c3gte	7B	Precision	0.0073	[−0.0293, 0.0414]	0.0505	0.0414	0.0079	null (undetected)
c4zscore	72B	F1	−0.0029	[−0.0162, 0.0052]	0.0153	0.0162	0.0122	null (undetected)
c4zscore	72B	Precision	−7.0e−04	[−0.0089, 0.0052]	0.0101	0.0089	0.0083	null (undetected)
c4zscore	7B	F1	−0.0162	[−0.0364, 0.0018]	0.0273	0.0364	4.0e−04	null (undetected)
c4zscore	7B	Precision	−0.0079	[−0.0193, 0.0028]	0.0158	0.0193	2.0e−04	null (undetected)
c5llama	Llama 70B	F1	0.0092	[−0.0137, 0.0379]	0.0369	0.0379	0.0548	null (bounded)
c5llama	Llama 70B	Precision	0.0150	[−0.0086, 0.0486]	0.0408	0.0486	0.0483	null (undetected)
c5llama	Llama 8B	F1	−0.0062	[−0.0285, 0.0155]	0.0314	0.0285	0.0436	null (bounded)
c5llama	Llama 8B	Precision	−0.0102	[−0.0290, 0.0078]	0.0264	0.0290	0.0385	null (bounded)
c6s43	72B	F1	0.0041	[−0.0022, 0.0121]	0.0102	0.0121	0.0349	null (bounded)

Configuration	Generator	Metric	Mean Δ	95% CI	MDE (80%)	Largest effect admitted	LLM reference	Verdict
c6s43	72B	Precision	0.0027	[−0.0015, 0.0084]	0.0070	0.0084	0.0290	null (bounded)
c6s43	7B	F1	−0.0155	[−0.0768, 0.0475]	0.0888	0.0768	0.0059	null (undetected)
c6s43	7B	Precision	0.0075	[−0.0429, 0.0661]	0.0779	0.0661	0.0049	null (undetected)
c6s44	72B	F1	0.0044	[−0.0084, 0.0151]	0.0168	0.0151	0.0604	null (bounded)
c6s44	72B	Precision	0.0055	[−0.0027, 0.0130]	0.0112	0.0130	0.0497	null (bounded)
c6s44	7B	F1	−0.0259	[−0.0785, 0.0229]	0.0725	0.0785	0.0134	null (undetected)
c6s44	7B	Precision	0.0142	[−0.0334, 0.0676]	0.0722	0.0676	0.0094	null (undetected)
c7rate002	72B	F1	−0.0152	[−0.0364, −3.5e−04]	0.0258	0.0364	0.0036	negative
c7rate002	72B	Precision	−0.0054	[−0.0569, 0.0327]	0.0640	0.0569	0.0046	null (undetected)
c7rate002	7B	F1	−0.0394	[−0.0703, −0.0131]	0.0409	0.0703	0.0000	negative
c7rate002	7B	Precision	−0.0189	[−0.0922, 0.0429]	0.0965	0.0922	0.0000	null (undetected)
c7rate010	72B	F1	−0.0150	[−0.0353, 0.0021]	0.0267	0.0353	0.0081	null (undetected)
c7rate010	72B	Precision	0.0280	[−0.0081, 0.0681]	0.0545	0.0681	0.0337	null (undetected)
c7rate010	7B	F1	−0.0394	[−0.0744, −0.0088]	0.0469	0.0744	2.0e−04	negative
c7rate010	7B	Precision	0.0217	[−0.0315, 0.0801]	0.0798	0.0801	0.0020	null (undetected)
c8rec050	72B	F1	−0.0060	[−0.0204, 0.0067]	0.0194	0.0204	0.0130	null (undetected)
c8rec050	72B	Precision	0.0310	[0.0048, 0.0660]	0.0438	0.0660	0.0389	positive
c8rec050	7B	F1	−0.0118	[−0.0414, 0.0163]	0.0412	0.0414	0.0019	null (undetected)
c8rec050	7B	Precision	0.0639	[0.0109, 0.1263]	0.0824	0.1263	0.0031	positive
c8rec070	72B	F1	0.0063	[−0.0119, 0.0246]	0.0261	0.0246	0.0227	null (undetected)
c8rec070	72B	Precision	0.0148	[0.0013, 0.0301]	0.0206	0.0301	0.0296	positive
c8rec070	7B	F1	0.0178	[−0.0150, 0.0486]	0.0455	0.0486	0.0079	null (undetected)
c8rec070	7B	Precision	0.0388	[0.0090, 0.0710]	0.0443	0.0710	0.0065	positive

S18. Per-alarm latency of each stage

Measured on one DGX Spark (GB10) node with the same code paths and serving configuration as the main experiment. The reranking row includes the retrieval stage it depends on and is therefore not additive with it.

Stage	Unit	n	Mean	Median	p90	Notes
Retrieval index build (one-off)	s	1	27.11	27.11	27.11	
Retrieval (k=5)	ms	60	14.86	14.14	17.34	
Retrieval + cross-encoder rerank (pool 10→5); includes retrieval	ms	60	68.32	66.75	81.74	
LLM 7B (single alarm, retrieval arm)	s	40	2.93	2.87	3.46	

Stage	Unit	n	Mean	Median	p90	Notes
LLM 72B (single alarm, retrieval arm)	s	40	32.72	31.22	38.72	

S19. Coding of the RAG/LLM anomaly-detection strand against this study's two controls

The positioning claim in Section 2.6 is an absence claim, so its basis is recorded here. Every work cited in Section 2.1 appears in the table (13 coded, one excluded: the RAG formulation itself, which is not an anomaly-detection system). Coding was done from the full text where we hold it and is left undetermined otherwise — an abstract cannot establish that a study did *not* do something. Search terms were *ablation*, *w/o*, remove/disable a named component, *operating point*, and matched/common/shared recall or threshold; hit counts are reported so a reader can repeat the search. Of the 8 works read in full, 5 report a component-wise contrast and 0 hold a common operating point across the contrasted arms; the remaining 5 were available only in abstract and are not coded.

Work	Basis	Component contrast	Common operating point	Evidence	Search hits
Chabane2026	fulltext	not located	n/a	no search term returned a hit	—
Chen2026b	fulltext	yes	not located	Sect. 4.4 demonstrates the necessity of each key architectural component through a series of ablation studies	ablation=11
Duan2025	abstract only	—	—	—	—
Guo2026	abstract only	—	—	—	—
Liu2024a	fulltext	yes	not located	Ablation study. In this section, our goal is to investigate the role of each component in our method.	ablation=6; w/o variant=4
Maru2025	fulltext	yes	not located	contrasted against 'w/o training' (14 hits) and 'w/o SMA' (3 hits) variants	w/o variant=17
Pan2024	fulltext	not located	n/a	no search term returned a hit	—
Perera2025	fulltext	yes	no	Table III contrasts the autoencoder baseline (85th/90th/95th percentile thresholds) with the hybrid AE-RAGX arms (gpt-4o, gemini-2.5-pro)	—
RussellGilbert2025	fulltext	yes	no	Table II pairs a retrieval-free model with the retrieval one on SKAB (F1 0.56 against 0.74)	—
Soechit2025	abstract only	—	—	—	—
Yang2024	fulltext	not located	n/a	'ablation' 0 hits, 'threshold' 0 hits, 'operating point' 0 hits; the 4 'w/o' hits are not component ablations (e.g. 'recommend effective AD models w/o relying on historical performance data')	w/o variant=4
Zhang2024	abstract only	—	—	—	—
Zhang2025e	abstract only	—	—	—	—
